# Spatially resolved mapping of histones reveals selective neuronal response in Rett syndrome

**DOI:** 10.1111/febs.70534

**Published:** 2026-04-01

**Authors:** Frederike Schäfer, Giuseppina De Rocco, Moritz Völker‐Albert, Ignasi Forne, Nicoletta Landsberger, Axel Imhof, Shibojyoti Lahiri

**Affiliations:** ^1^ Biomedical Center Munich, Department of Molecular Biology, Faculty of Medicine Ludwig‐Maximilians Universität München Planegg‐Martinsried Germany; ^2^ San Raffaele Rett Research Unit, Neuroscience Division IRCCS San Raffaele Scientific Institute Milan Italy; ^3^ Department of Medical Biotechnology and Translational Medicine, Faculty of Medicine University of Milan Italy; ^4^ MoleQlar Analytics GmbH München Germany; ^5^ Biomedical Center Munich, Protein Analysis Unit (ZfP), Faculty of Medicine Ludwig‐Maximilians Universität München Planegg‐Martinsried Germany

**Keywords:** histone modifications, laser capture microdissection, mass spectrometry imaging, Mecp2, Rett syndrome, spatial proteomics

## Abstract

Rett syndrome (RTT), a severe neurological disorder caused by loss‐of‐function mutations in the X‐linked *Mecp2* gene, results in profound life‐long neurological dysfunction. Rett syndrome patients live an apparently normal initial life until 12–18 months of age, after which a progressive accumulation of a wide range of phenotypic manifestations sets in. While MeCP2 is known to regulate chromatin, its impact on global histone composition and dynamics remains poorly understood. Here, we combine mass spectrometry imaging (MSI) and laser capture microdissection (LCM) coupled to LC–MS/MS to systematically profile histone proteoforms in three key brain regions: the dentate gyrus (DG) and cornu ammonis (CA) of the hippocampus, and the cerebellum (Cb). Our analysis reveals striking neuron‐specific differences in histone composition between Mecp2‐deficient and wild‐type (WT) mice. Interestingly, the expression of a pathogenic Mecp2 missense mutant (Y120D) results in subtler changes in histone composition that are distinct from the null mutation. This study provides the first spatially resolved epigenetic atlas of histone proteoforms in Rett syndrome and suggests that Mecp2 loss perturbs chromatin homeostasis in a neuron‐ and mutation‐dependent manner. Our findings underscore the critical need for cell‐type‐resolved analyses to unravel the mechanistic underpinnings of Rett syndrome and emphasize the importance of personalized therapeutic strategies that consider both the affected cell type and particular Mecp2 mutation.

## Graphical abstract

Rett syndrome (RTT) is a severe neurological disorder caused by loss‐of‐function mutations in the X‐linked *MECP2* gene that causes profound life‐long neurological dysfunction. MeCP2 regulates chromatin, yet its influence on histone composition and dynamics is unclear. Combining MALDI‐MSI with LCM–LC‐MS/MS, we mapped histone proteoforms across dentate gyrus, cornu ammonis, and cerebellum in two mouse models of RTT. Mecp2 loss‐of‐function elicited region‐ and mutant‐specific alterations in the histone proteoform landscape, affecting total histone levels and post‐translational modifications. This study provides the first spatially resolved epigenetic atlas of histone proteoforms in RTT and suggests that Mecp2 loss perturbs chromatin homeostasis in a neuron‐ and mutation‐dependent manner.

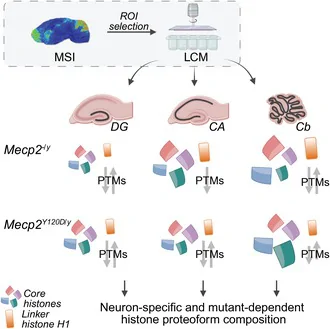

**Abbreviations**
ACN: acetonitrile
CA: Cornu Ammonis
Cb: cerebellum
CNS: Central Nervous System
DG: dentate gyrus
FDR: false discovery rate
GO: gene ontology
HE: Hematoxylin and Eosin
HDAC: histone de‐acetylase
HEPES: 4‐(2‐hydroxyethyl)‐1‐piperazineethanesulfonic acid
ITO: indium tin oxide
LC–MS/MS: liquid chromatography coupled tandem mass spectrometry
LCM: laser capture microdissection
LIMMA: linear models for microarray data
MALDI‐MSI: Matrix‐Assisted Laser Desorption/Ionization‐Mass Spectrometry Imaging
MBD: methylated‐DNA binding domain
Mecp2: methylated‐DNA binding Protein 2
PCA: Principal Component Analysis
ROI: regions of interest
RTT: Rett Syndrome
SA: Sinapinic acid
SIL: Stable Isotope Label
TCA: tricholoroacetic acid
TIC: total ion count
TFA: trifluoroacetic acid
TRD: transcription repression domain
WT: wild type

## Introduction

Rett syndrome (RTT) is a disorder of the central nervous system (CNS) that primarily affects females and is the second most common cause for intellectual disability in females after Down syndrome [1]. Children with RTT typically exhibit apparently normal postnatal development during the first 12–18 months of life, after which a progressive neurological decline ensues. Hallmarked by escalating motor, speech, and cognitive impairments, the situation is further complicated by the progressive occurrence of several other symptoms such as scoliosis, seizures, respiratory dysrhythmias, ataxia, dysphagia, and gastrointestinal problems, reflecting widespread and evolving neurological dysfunction [2, 3].

RTT is caused by loss‐of‐function mutations in the X‐linked gene encoding methyl‐CpG‐binding protein 2 (*MECP2*) [4, 5]. Most of the missense mutations in *MECP2* that correlate with severity of symptoms have been found in either the methyl binding domain (MBD) and/or the transcription repression domain (TRD) [2]. MeCP2 was initially identified as a transcriptional repressor that recruits co‐repressor complexes containing histone de‐acetylases (HDACs) [6, 7]. However, additional studies showed that MeCP2 can also affect gene expression through interactions with transcriptional activators [8, 9]. Beyond its role in the direct regulation of transcription, MeCP2 has also been reported to regulate global chromatin structure [8, 10, 11, 12, 13, 14]. It induces chromocenter clustering [15], maintains chromatin loops [16] and nucleosome‐free regions [17] and compacts nucleosomal arrays both *in vitro* and *in* vivo [11, 18, 19]. Mecp2 is highly expressed in mature neurons where it partly replaces linker histone H1 [20, 21]. Loss of Mecp2 leads to an approximately 2‐fold increase in linker histone H1 in neurons suggesting an overlapping set of chromatin‐associated functions [19, 22]. It has been shown previously that loss of Mecp2 leads to diverse physiological effects depending on the type of affected neurons [23, 24, 25, 26, 27, 28, 29, 30]. There are two different yet non‐exclusive hypotheses for why a specific knockout results in different physiological outputs. On one hand, deletion of the *Mecp2* gene may disrupt a core function shared across neurons, with phenotypic differences arising simply from the distinct roles of the affected brain regions. On the other hand, cell‐type‐specific studies reveal that Mecp2 deficiency alters diverse gene sets in different neuronal populations suggesting that Mecp2's molecular role is highly context‐dependent. Resolving these models requires spatial analysis of molecular changes upon *Mecp2* removal *in situ* to investigate whether regional phenotypes stem from uniform dysfunction or cell‐type‐specific mechanisms. In addition, expression of *Mecp2* is spatio‐temporally regulated making it crucial to study the effects of its loss in the spatial context [31, 32].

Histone proteoforms—including canonical histones, their variants, and post‐translationally modified forms—play a critical role in regulating chromatin architecture and shaping the epigenetic landscape in neurons [33, 34, 35, 36, 37, 38, 39]. As a result, they have been associated with or implicated in several neurodegenerative diseases [40, 41, 42, 43, 44]. Mecp2, as a chromatin‐associated protein, is widely believed to influence these structural and regulatory processes [10, 11, 12, 17, 39, 45]. Therefore, we wanted to comprehensively study the effect of Mecp2 loss‐of‐function on the histone composition *in situ*. So far, there have been few studies investigating the effects of Mecp2 loss on histone proteoforms. These studies, however, were restricted by the availability of specific antibodies, lack of spatial context and a comprehensive understanding of the entire histone proteoforms landscape [45, 46, 47, 48, 49]. To address this gap, we used a dual spatial approach that involves mass spectrometry imaging (MSI) and laser capture microdissection (LCM) coupled to LC–MS/MS. This strategy enabled unbiased, high‐resolution quantification of histone proteoforms across distinct brain regions. Based on preliminary observations and physiological context, we focused our measurements on the dentate gyrus granule cell layer (DG) and cornu ammonis pyramidal neurons (CA) of the hippocampus, and the cerebellar granule cell layer (Cb).

Mecp2 deficiency induces profound, cell‐type‐specific alterations in nucleosome composition, total histone levels, and modification patterns. We observe that the levels of all histone variants, including those of linker histone H1, are substantially disturbed in absence of Mecp2. In addition, lack of Mecp2 is also associated with major changes of global modification patterns across the neuronal subpopulations. These changes occur in the absence of significant alterations in the expression levels of histone‐modifying enzymes, implicating post‐translational mechanisms or enzyme redistribution as key drivers of the observed chromatin remodeling. We compared the findings with neuronal populations of a mouse model carrying the human pathogenic mutation Y120D [50]. The *Mecp2* ^*Y120D/y*^ model (KI) recapitulates RTT pathology and exhibits distinct chromatin dynamics to those observed in Mecp2 knockouts (KO) [50, 51]. We observe that the effects on histone proteoforms in the knock‐in model are discordant to those of the knockout, indicating the possibility of distinct molecular pathways underlying different mutations for RTT.

Taken together, our work provides a spatially resolved atlas of histone proteoforms in WT mice and their alterations in RTT. We highlight the importance of analyzing chromatin in a spatially resolved manner. By linking Mecp2 loss‐of‐function (both in null and mutant models) to discrete histone proteoform alterations, we provide novel insights into the possible epigenetic mechanisms driving RTT associated with different Mecp2 mutations.

## Results

### Spatially resolved quantification of histone proteoforms

To investigate Mecp2 loss‐of‐function–induced molecular alterations in both Mecp2 null (Mecp2^−/y^) and Mecp2 mutant (Mecp2^Y120D/y^) brains *in situ*, we employed mass spectrometry imaging (MSI) to spatially resolve intact proteoforms in an unbiased, high‐throughput manner (Fig. 1A). This approach allowed extensive MS1‐based filtering of intact protein masses, thereby contributing to the rapid screening method. A relatively narrow range of masses for intact histones combined with their correlation with the respective nuclear density allows for direct assignment of histone proteoforms [52]. This approach enabled a proteome‐wide assessment of regional molecular alterations in RTT without prior target selection. Unsupervised clustering of MSI signals revealed that hippocampal neurons (DG and CA regions), cortex, and cerebellar granule layer (Cb) formed distinct spatial clusters early in the analysis (Fig. 1B; see dendrogram). Principal component analysis (PCA) confirmed the clear separation among the exclusive neuronal populations (Fig. 1C), primarily driven by strong histone signals (Fig. S1A; Table S1) as a result of high nuclear density [52].

**Fig. 1:**
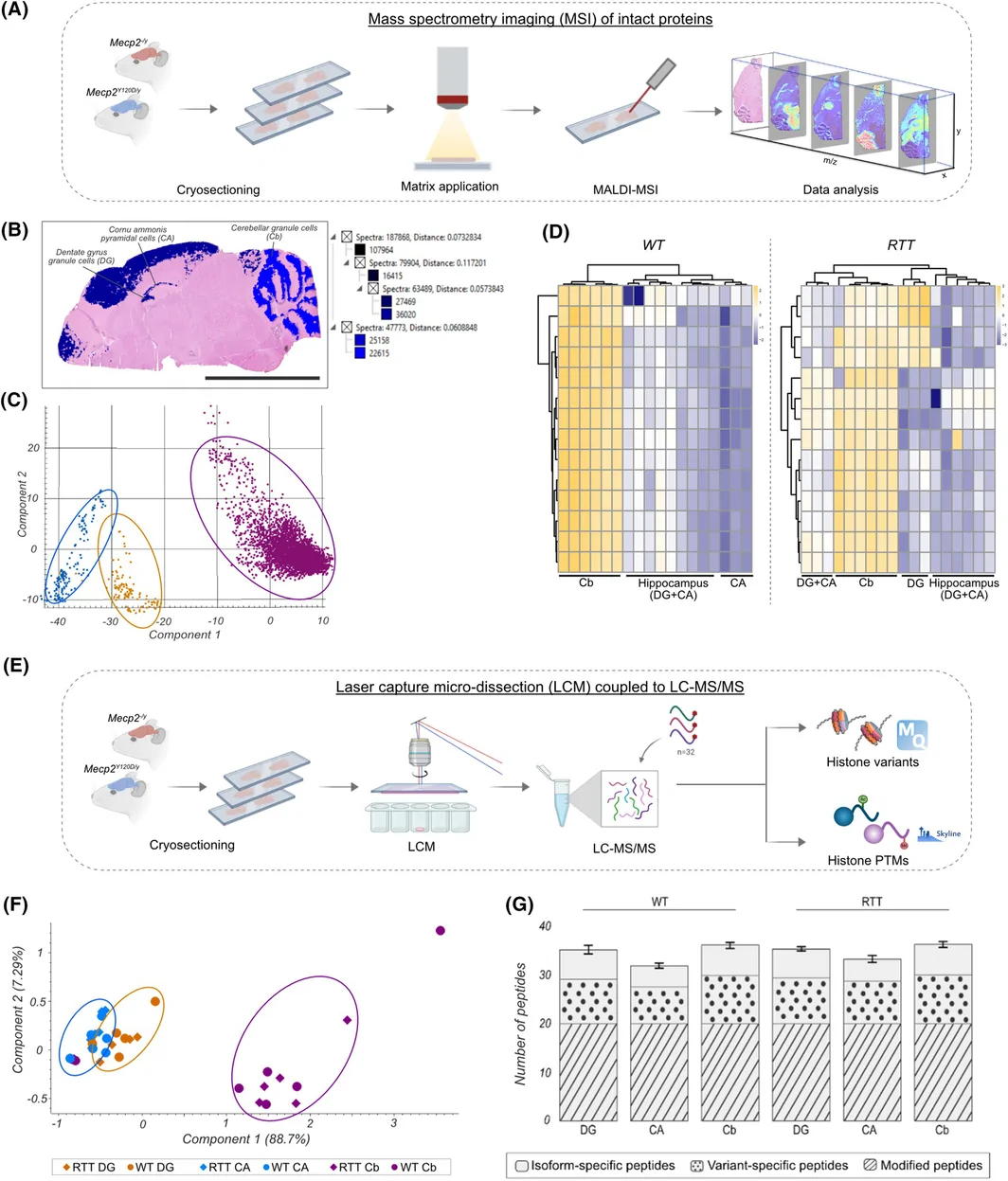
Rapid spatial screening reveals altered histone proteoforms. (A) Workflow for MALDI‐MSI. After cryosectioning, brain sections are sprayed with matrix and analyzed using rapifleX tissuetyper, followed by data analysis in flexImaging and SCiLS Lab. (B) Hierarchical clustering of MSI data reveals early separation of cortex/hippocampus (darkblue) and cerebellum (blue). Dendrogram of unbiased clustering analysis. Scale bar = 5 mm. (C) PCA of MSI data. Dots represent individual pixels. Neuronal populations of CA (blue), DG (orange) and Cb (pink) can be separated based on their proteomic signature generated by MALDI‐MSI. (D) Unbiased clustering of MSI‐detected histone proteoforms in WT and RTT model brains. Histone identities were assigned to masses based on previous work [50]. Each column represents a biological replicate of WT, *Mecp2* ^*−/y*^ and *Mecp2* ^*Y120D/y*^. (E) Workflow for laser capture microdissection (LCM) coupled to LC–MS/MS. Mouse brains were cryosectioned and regions of interest excised by LCM. Histone extraction was performed on collected tissue samples with the addition of 32 stable isotope labeled (SIL) peptides. Intact histones were identified and quantified by MaxQuant and modified peptides were manually picked and quantified in Skyline. (F) PCA of LC–MS/MS data of basic proteins. These proteomic signatures are sufficient to separate the neuronal population DG, CA, and Cb. (G) Number of detected peptides of canonical histone isoforms, variants and modified peptides are reproducible across sample groups with >30 proteoforms. Error bars = SEM. *n* = 3 (*n* = 2 for *Mecp2* ^*−/y*^).

Fourteen masses were assigned to members of the histone families. Histones H1 and H4 are mostly detected in their modified variants. Seven masses can be attributed to the histone H2A/B families but isoform assignment is imprecise due to close mass similarity and lack of clear spectral separation and mass fragmentation (Fig. 1D). While the overall clustering patterns appeared similar between Mecp2‐deficient and WT brains, we observed pronounced differences in the composition of histone proteoforms (Fig. 1D, Table S2 [52]). Notably, the clear separation of cerebellar and hippocampal regions seen in WT brains was disrupted in their RTT counterparts, indicating altered composition of histones in these areas (Fig. 1D). Given the known physiological relevance of these brain regions to RTT pathogenesis (Fig. S1B), and their emergence through unbiased *in situ* histone proteoform screening, we microdissected the identified regions (DG, CA, and Cb) using laser capture microdissection (LCM) and performed LC–MS/MS analysis to achieve high‐resolution, exhaustive, and precise identification and quantification of histone proteoforms (Fig. 1E). This integrative spatial‐proteomic strategy allowed us to resolve the region‐specific histone landscape potentially underlying the RTT phenotype. For the LC–MS/MS approach, histones were acid extracted and subsequently analyzed using a tailored protocol for quantitation of histone variants and modified residues (Fig. 1E) [53, 54, 55, 56, 57]. Based on the extracted ‘proteome’, our regions of interest clustered independently within the principal component space, with the hippocampal populations expectedly closer together than the cerebellum (Fig. 1F). Furthermore, clustering using only histone‐derived peptides indicates genotypic separation within the respective regions, highlighting the substantial contribution of histones to the overall clustering (Fig. S1C). Our protocol was sensitive enough to quantify more than 30 different variant‐specific as well as modified peptides from a minimum of 30 000–35 000 cells (from CA). We identified a minimum of 11 different core and linker histone isoforms or variants and profiled 20 modifications on H3/H4 in WT and RTT brains (Fig. 1G).

### Loss of Mecp2 induces variance in histone levels

#### Canonical histones

Our approach allowed us to precisely quantify the amount of histones in WT and *Mecp2* ^*−/y*^ brains. As extraction efficiency may influence histone abundance, we used co‐extracted non‐histone proteins to control for this type of technical variation. The vast majority of extracted proteins is unaffected by the loss of Mecp2 (Fig. S2A,B). To account for potential differences in genomic material that could possibly influence histone amounts, the intensities of the individual peptides were normalized to the total amount of DNA present in the respective samples (Fig. S2C). To quantify the total level of core histones we used peptides that were detected in all replicates and all respective isoforms (residues 40‐49 for H3, 47‐56 for H4, 22‐30 for H2A and 101‐121 for H2B). Looking at region‐specific histone alterations, we observe opposing trends within the neuronal populations of the hippocampus (Fig. 2A). While DG shows a moderate decrease in levels of all core histones, CA is associated with minor upregulation of the same. Strikingly, cerebellar granule cells show an imbalance of histone amounts with an increase of H3 and H2B and a reduction in H2A and H4 (Fig. 2A) levels. This disparity in histone stoichiometry indicates towards a considerable fraction of non‐DNA bound histones within the cells, the function of which is currently unclear.

**Fig. 2:**
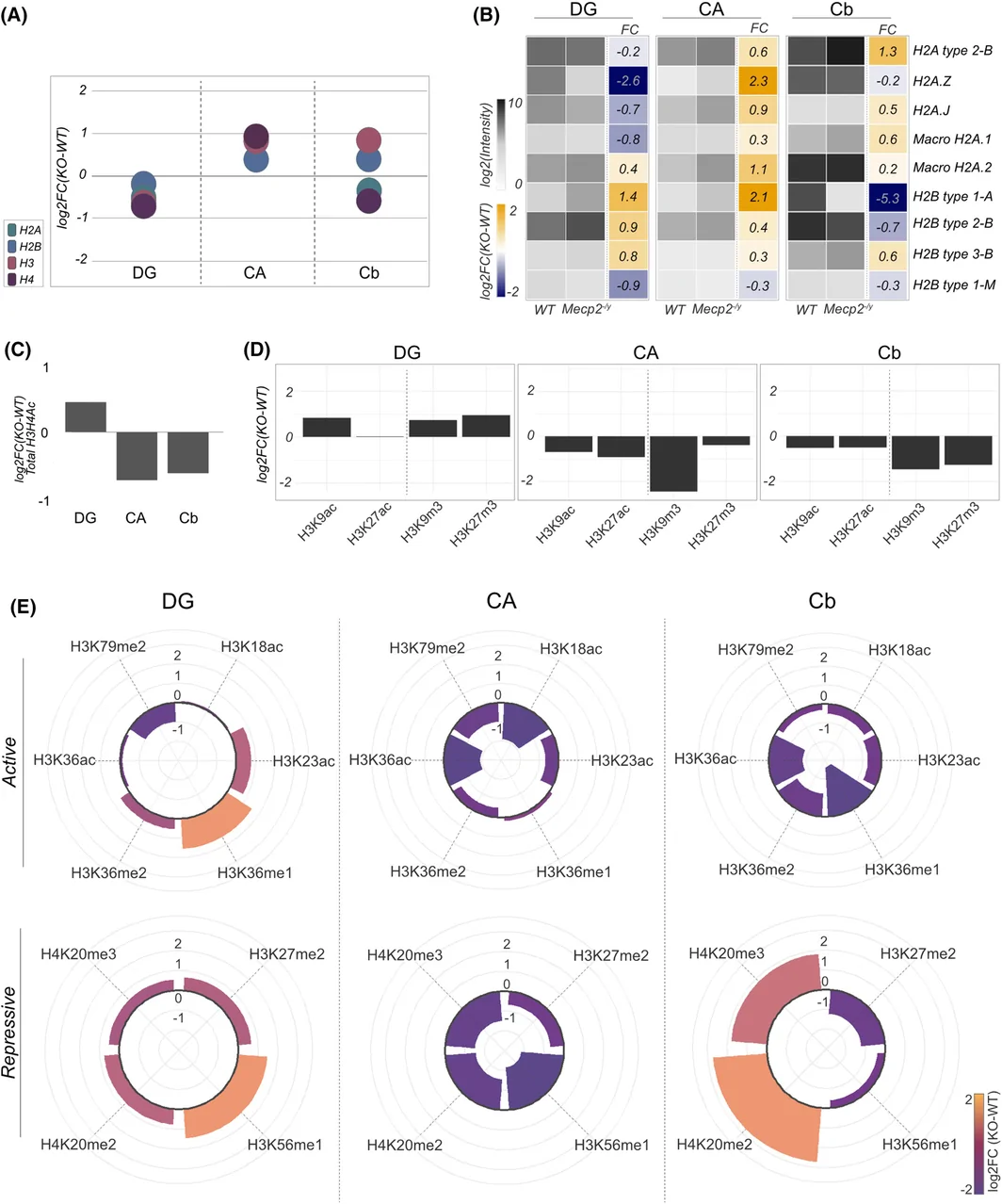
Neuronal histone proteoform landscape is altered by loss of *Mecp2*. (A) Alteration of total histone abundance upon loss of *Mecp2* in neuronal populations DG, CA, and Cb in log_2_ fold change. (B) log_2_ intensities of H2A/B peptides in WT and *Mecp2* ^*−/*y^ brains and respective fold changes in DG, CA, and Cb. (C) Fold change of total acetylation of H3/H4. (D) Fold change of H3K9/K27 acetylation (ac) and tri‐methylation (me3) in neuronal populations DG, CA, and Cb. (E) Fold change of histone post‐translational modifications associated with active and repressive transcription. Fold changes between KO and WT were calculated from log_2_‐transformed normalized intensities of modified peptides. *n* = 3 (*n* = 2 for *Mecp2* ^*−/y*^).

#### Histone variants and isoforms

The core histone proteins are also characterized by the presence of variants that have distinct functional roles including regulation of neuronal functionality [33, 34, 36, 37, 38]. Therefore, change of relative proportions of these variants might play a crucial role in brain functionalities, particularly in the absence of key chromatin modulator Mecp2. Histone H3.3 is one of the most important histone variants in the adult mammalian brain, as it constitutes majority of the H3 pool in post‐mitotic cells [33, 34, 58]. Similar to previous findings [34], we observe that H3.3 makes up the majority (50‐60%) of the total H3 pool in hippocampal neuronal populations, while the contribution is lower in Cb (Fig. S3A) of Mecp2 null mice. This relative proportion of the H3.3 variant remains unaffected in granule layers of Mecp2 null mice but is reduced in pyramidal neurons (Fig. S3A).

All H2A/B variants and isoforms were quantified based on corresponding unique peptides (Fig. S3B; Files S1, S2) detected across most samples. Expectedly, WT brains are characterized by high expression of the canonical H2A type 2‐B and macroH2A.2 across all neuronal populations [59] (Fig. 2B). In addition to these, granule layers of WT DG and Cb show high abundance of H2A.Z. Relative contribution of H2A.J to the total H2A pool is more dominant within the hippocampus as compared to the Cb (Fig. 2B). Upon loss of Mecp2, different variants and isoforms are altered in a cell‐type‐specific manner (Fig. 2B). Most noteworthy is the strong up – and downregulation of H2A.Z in CA and DG, respectively, followed by H2A.J and macroH2A.1, albeit to a lesser extent. Interestingly, except for macroH2A.2, all other H2A variants show an opposite trend of dysregulation within the two hippocampal neuron populations (Fig. 2B). In the *Mecp2* ^*−/y*^ Cb, however, H2A.Z stands out in being slightly downregulated as compared to all other H2A variants (Fig. 2B). While canonical H2A still remains the most abundant isoform in KO neurons, relative contribution of variants is distinct to that of WT, suggesting neuron‐specific compositional changes of the cellular H2A pool (Fig. 2B).

For H2B, we primarily detect canonical H2B isoforms that are located either within the major histone cluster on chromosome 13 (H2B type 1A and H2B type 1 M) or on the minor clusters on chromosome 3 (H2B type 3B) or 11 (H2B type 2B) (Fig. 2B) [60, 61]. Similar to H2A, loss of Mecp2 induces regional re‐organization of the H2B pool, as indicated by distinct alterations and relative abundances of H2B isoforms across different regions of the *Mecp2* ^*−/y*^ brains (Fig. 2B). Most affected by this rearrangement is H2B type 1‐A, which displays the highest degree of change across all three regions (Fig. 2B).

To determine if the aforementioned dysregulation in histone abundances in RTT originates at the transcript level, proteome‐level observation was compared with publicly available total RNA‐seq data of WT and *Mecp2* ^*−/y*^ brains (Fig. S3C [62, 63, 64, 65, 66, 67]). For this purpose, we utilized the resources provided by MeCP2pedia alongside publicly available data for the relevant cell types [49, 62, 63, 64, 65, 66, 67]. Nevertheless, none of the available RNA‐seq datasets include complete histone profiles, preventing validation of all detected proteoforms at the transcript level. For the identifiable transcripts, Mecp2 loss did induce corresponding changes, although the magnitude and direction varied considerably across studies.

For the hippocampal neuronal populations DG and CA, trends for most histones were largely concordant between the transcript and protein levels, except H2B type 2‐B and type 1‐M (Fig. S3C [62, 63, 64, 65, 66, 67]). Variability was most pronounced for Cb, both across transcriptome and proteome datasets. Consistent with our proteomic measurements, an increase in total H3 and H2A type 2‐B and a decrease in total H4 and H2B type 1‐M transcripts were observed. However, for macroH2A.2 and H2B type 2‐B, trends observed at the proteome level matched with only one study. Interestingly, H2A.Z and H2A.J showed opposite trends at the proteome and transcriptome level in Cb (Fig. S3C [62, 63, 64, 65, 66, 67]).

#### Core histone modifications

Mecp2 is known to co‐recruit histone deacetylases (HDACs) as part of a repressor complex, yet reports on its impact on global histone acetylation are inconsistent [22, 47]. While some studies show increased H3 acetylation in neuronal nuclei from mouse brain [22], others report no change in clonal cell cultures from RTT patients, which instead exhibit hyperacetylation of H4 [47]. To resolve these apparent discrepancies, we aimed to systematically analyze total histone acetylation in a cell‐type‐specific manner within the RTT brains. In *Mecp2* ^*−/y*^ brains, we observed only DG granule cells to be associated with an overall increase in total H3/H4 acetylation, whereas the other two investigated neuronal populations show a global depletion of the acetylation mark (Fig. 2C). However, this does not seem to be facilitated by altered levels of Mecp2‐recruited modifiers, as we do not see changes in overall HDAC abundance (Fig. S4A).

Two most prominent class of active and repressive marks studied in the context of Mecp2‐chromatin interaction are the tri‐methylation (me3) and acetylation (ac), specifically of the K9 and K27 residues of core histone H3 [45, 48, 49]. However, the impact of Mecp2 loss on these epigenetic modifications remains unclear, particularly globally and in terms of cell‐type‐specific patterns that may underlie the pathophysiology of RTT. Our unbiased approach enabled us to simultaneously profile these modifications within the neuronal populations of interest (File S3). WT and *Mecp2* ^*−/y*^ brains display a unique histone modification landscape, independent of total histone levels.

The relative contribution of individual marks, expressed as the ratio of each modified peptide to all other forms of the same peptide, varies substantially (Fig. S4B) in Mecp2 null brains. This gives valuable insight into contributions of individual histone modifications within a particular genotype. As histone amounts themselves are subjected to change, our aforementioned analysis correct for that and allow us to compare the modification levels directly between genotypes. We observe that the trends of dysregulation of these modifications follow a cell‐type‐specific pattern (Fig. 2D). Upon loss of Mecp2, both the acetylation (ac) and tri‐methylation (me3) marks of H3K9 and H3K27 are enriched in the DG, whereas they are depleted in the other two neuronal populations, albeit to quite different extents (Fig. 2D). These findings likely reflect the ambivalent nature of Mecp2 function, having both repressive and activating effects on local gene expression. Our approach captures an accumulation of local dysregulation of histone proteoforms across different neuronal populations, revealing cell‐type‐specific selectivity as an additional layer of epigenetic dysregulation in RTT beyond gene‐specific alterations.

Additionally, many other active and repressive histone marks (outside of H3K9/27 me3/ac). In *Mecp2* ^*−/y*^ DG, the chromatin is characterized by an enrichment of active gene expression marks such as H3K23ac and H3K36me1/2 along with a global gain of repressive marks (Fig. 2E, left panel). However, the CA and Cb exhibit an overall depletion of these marks except for H3K36me1 in CA and H4K20me2/3 in Cb (Fig. 2E, middle and right panel). Notably, these alterations of modified peptides are anti‐correlated to changes in total H3/H4 levels in the respective cell populations, indicating preferential gain/loss of unmodified proteoforms (Fig. 2A; Fig. S4C).

The observed alterations in the chromatin landscape, as reflected by changes in histone modifications, are not facilitated by differential regulation of the corresponding modifiers, which either remain consistent across cell types or the direction of dysregulation doesn't align with the effect of the modification it drives (Fig. S4A). This suggests that the dysregulation arises most likely from altered enzymatic activity and/or impaired recruitment to chromatin.

Taken together, loss of Mecp2 function induces complex changes in the histone modification landscape that are unique for specific neuronal populations. The observed alterations likely reflect local changes in chromatin state that vary in magnitude and pattern within each neuronal subtype, highlighting the context‐dependent nature of epigenetic dysregulation in *Mecp2* ^*−/y*^ brains.

### Alterations of linker H1 levels and variant composition

Elevated histone H1 expression in RTT models is regarded as a potential compensation for Mecp2 loss‐of‐function due to its increased deposition on chromatin in neurons of *Mecp2* ^*−/y*^ mice. However, the underlying mechanisms and its generality remain elusive due to conflicting evidence [19, 20, 21, 22].

Our approach allowed us to profile the total H1, including all H1 variants, comprehensively across the neuronal populations. For accurate quantification, one shared peptide among all H1 variants was taken into consideration to approximate for total H1 levels. We indeed observe an increase of total H1 in Cb, whereas the two hippocampal neuronal populations show different trends (Fig. 3A). While H1 levels increase modestly in CA, they are reduced in *Mecp2* KO DG (Fig. 3A).

**Fig. 3:**
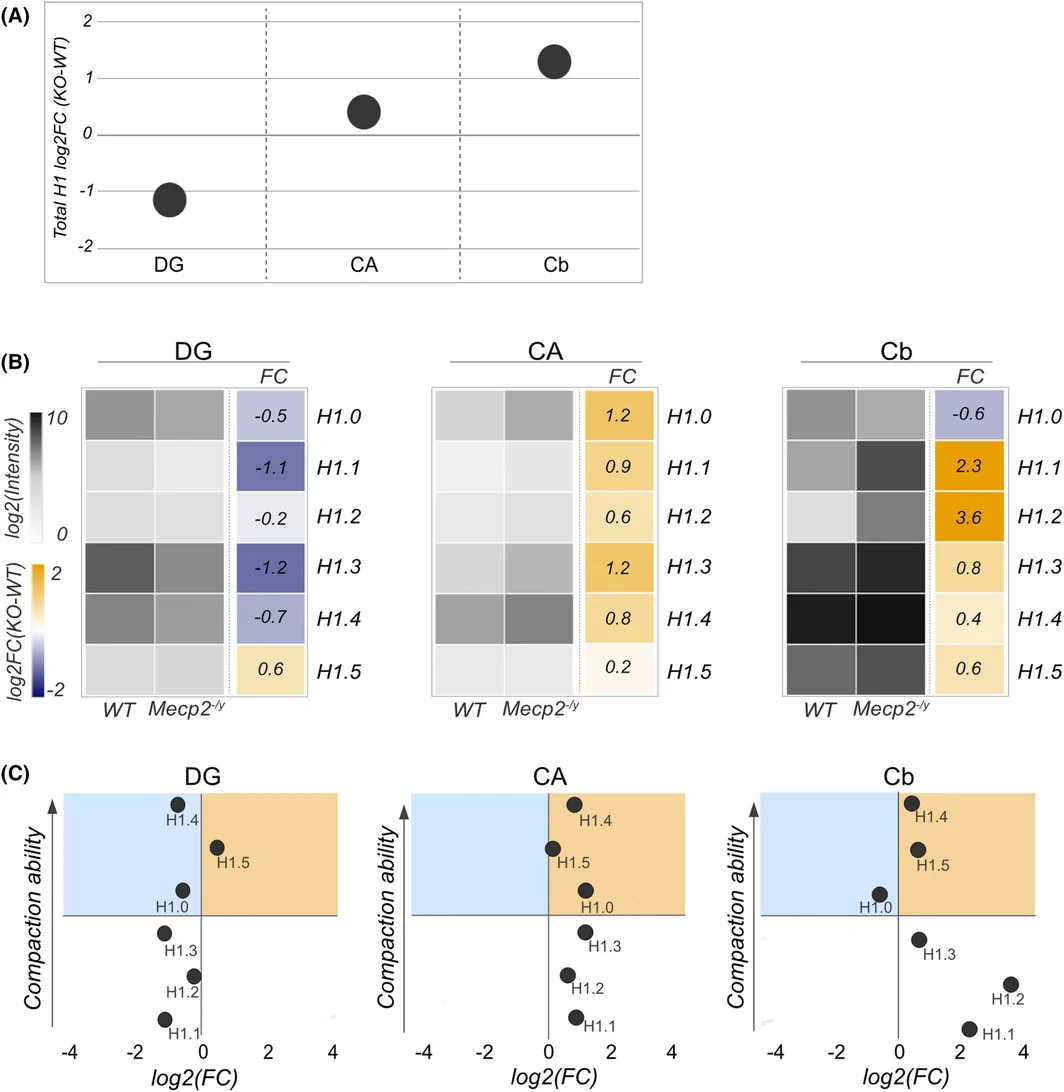
Histone H1 is affected by *Mecp2* loss in a neuron‐specific manner. (A) Total abundance of H1 in WT and *Mecp2* ^*−/y*^ in log_2_ fold change. (B) log_2_ intensities of H1 variant peptides in WT and *Mecp2* ^*−/y*^ brains and respective fold changes in DG, CA, and Cb. (C) Fold changes of histone H1 in correlation to their compaction ability. Compaction ability was scored based on previous classification by Prendergast and Reinberg [68]. Positive (orange) and negative (blue) fold changes of variants with high compaction ability are highlighted. *n* = 3 (*n* = 2 for *Mecp2* ^*−/y*^).

Considering that mammalian H1 has 7 somatic variants and relative expression of these variants possibly vary between different cell types [68, 69], we attempted to understand the relative dynamics of H1 variants upon Mecp2 loss. The applied methodologies allow us to quantify the relative contribution of different H1 variants to the total H1 pool based on one unique peptide characteristic to each variant that was detected across samples. Information about the unique peptides and their respective positions within the protein are provided in Fig. S4D and Files S1, S2. As expected, H1.4 and H1.0 are the predominant H1 variants across all WT neuronal populations [68], with the addition of H1.3 in granule cells (Fig. 3B). These variants are affected differently by loss of Mecp2, resulting in an altered composition of the H1 pool in *Mecp2* ^*−/y*^ brains. Overall, within the hippocampal populations of *Mecp2* KO, we observe that most H1 variants show a decrease in DG, while the trends are opposite in CA with the exception of H1.5 (Fig. 3B). Nonetheless, H1.0, H1.3 and H1.4 remain the variants with the most prominent contribution to the total H1 pool, despite different absolute levels compared to WT (Fig. 3B). A mixed effect characterizes the Cb, where the replication‐dependent variants H1.1‐H1.5 increase while H1.0 is depleted (Fig. 3B). This further reduces the proportion of H1.0 to the H1 pool in Cb, while H1.1 and H1.2 now substantially contribute to the pool as compared to the WT (Fig. 3B). This reflects the cell‐type‐specific contribution of individual somatic H1 variants and suggests against inferring just from total H1 levels and any one type of brain cells.

The degree of influence of different H1 variants on chromatin compaction depends on their differential chromatin binding affinity and compaction ability [68]. The fold change of individual variants between the WT and *Mecp2* ^*−/y*^ brains provides an indication about possible H1‐mediated chromatin compaction states in RTT (Tables S3 [68], S4). In *Mecp2* ^*−/y*^ DG, variants with a higher compaction ability are not particularly enriched (Fig. 3C, left panel). The strongest compactor H1.4 shows an enrichment in CA along with substantial enrichment of variants having a moderate compaction ability (Fig. 3C, middle panel). However, in Cb, the enrichment of H1 variants includes both the strongest compactors in the form of H1.4 and variants (H1.1 and H1.2) with lower compaction ability (Fig. 3C, right panel).

### Effects on histone proteoforms are mutant‐specific

Comprehensive characterization of histone proteoforms in the *Mecp2* KO mice reveals that Mecp2 loss has a profound and cell‐type‐specific effect on the histone composition. However, total absence of Mecp2 might lead to compensatory mechanisms within the brain. We therefore characterized a mouse model harboring the pathogenic mutant *Mecp2* ^*Y120D/y*^ that has previously been shown to have discordant chromatin features in spite of having similar phenotype [50].

Unlike *Mecp2* ^*−/y*^ animals, total histone levels change only marginally in the hippocampus (DG and CA), while cerebellar granule cells display an increase of H2A/B only (Fig. 4A). With respect to core histone variants, we observe that the effects have a cell‐type‐specific signature that is different from that of KO animals (Figs 2B, 4B). WT mice carry the canonical H2A type 2‐B as the major H2A isoform, followed by macroH2A.2 and H2A.J (Fig. 4B). Unlike the KO animals, the contribution of H2A.Z is considerably enriched in *Mecp2* ^*Y120D/y*^ hippocampus, whereas it is unchanged in the Cb (Fig. 4B). Interestingly, as opposed to *Mecp2* ^*−/y*^ Cb, the knock‐in animals display a marked depletion of both macroH2A.2 and H2A.J. This depletion is accompanied by a substantial enrichment of the canonical H2A type 2‐B contribution, resulting in a notable reduction in the variety of the H2A pool (Fig. 4B). H2B isoform expression and contribution differ between WT and *Mecp2* KI mice, displaying isoform‐specific alterations across all neuronal populations (Fig. 4B) that are different from their KO counterparts (Fig. 2B).

**Fig. 4:**
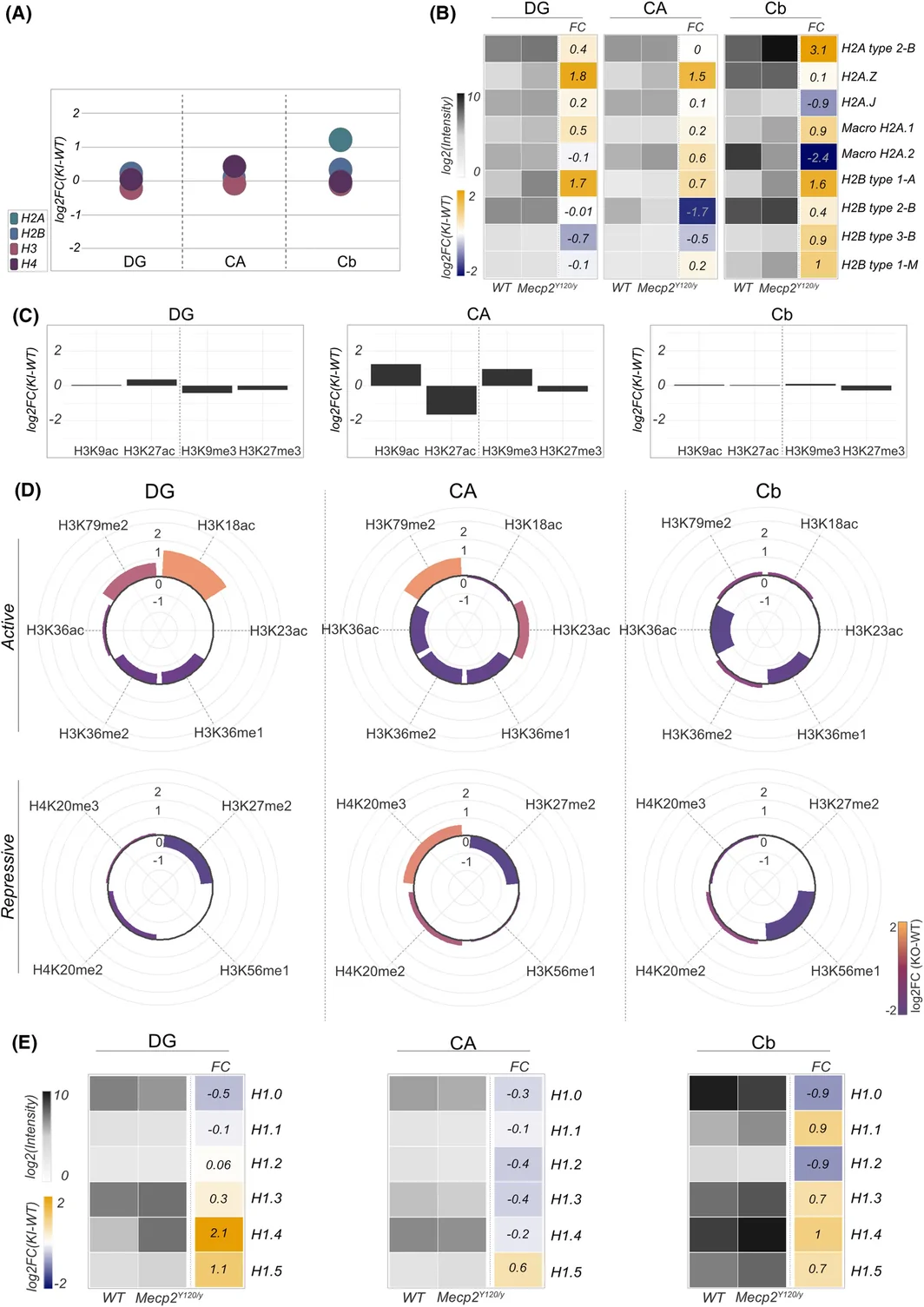
*Mecp2* ^*Y120D/y*^ induces distinct changes in neuronal histone proteoform levels. (A) Alteration of total histone abundance in neuronal populations DG, CA, and Cb of *MeCP2* ^*Y120D/y*^ brains. (B) log_2_ intensities of H2A/B peptides in WT and *Mecp2* ^*Y120D/y*^ brains and respective fold changes in DG, CA, and Cb. (C) Fold change of H3K9/K27 acetylation (ac) and tri‐methylation (me3) in neuronal populations DG, CA, and Cb. (D) Fold changes of histone post‐translational modifications associated with active and repressive transcription show unique trends across the cell types. Fold changes between KO and WT were calculated from log_2_‐transformed normalized intensities of modified peptides. (E) log_2_ intensities of H1 variant peptides in WT and *Mecp2* ^*Y120D/y*^ brains and respective fold changes in DG, CA, and Cb. *n* = 3 (*n* = 2 for *Mecp2* ^*−/y*^).

The Y120D mutation also has distinct effects on the deposition of histone modifications. Global H3/H4 acetylation levels are largely unchanged in DG, while they show a subtle increase in CA and a decrease in Cb and therefore differ substantially from KO brains (Fig. S5A). The histone modification landscape of the WT littermates is comparable to *Mecp2* ^*−/y*^ WT littermates, despite minor deviations in the relative abundances of single modified peptides. However, in mutant animals the composition of modified peptides is notably different (Figs S4B, S5B–D). *Mecp2* ^*Y120D/y*^ CA and Cb only show minor changes in the relative abundance of modified peptides. DG, on the other hand, shows a particularly high proportion of acetylated histone H3 peptides (Fig. S5B).

Here, the presence of the mutant protein has minimal effects on H3K9 and H3K27 tri‐methylation (me3) and acetylation (ac) in granule cell layers of both DG and Cb (Fig. 4C, left and right panel). In CA, the marks on H3K9 are enriched while H3K27 is depleted, particularly affecting H3K27ac (Fig. 4C, middle panel), thus deviating substantially from the situation in *Mecp2* ^*−/y*^ brains. Global effects on other active and repressive histone modifications show a cell‐type‐specific signature that is distinct from *Mecp2* ^*−/y*^ brains as well (Fig. 4D). Hippocampal neurons tend to gain H3K79me2 and H3K18/K23ac, which is accompanied by a loss of H3K27me2. In addition, all neuronal populations exhibit reduced levels of modified H3K36 (Fig. 4D). These alterations of the histone modification landscape differ vastly both in trend and amplitude from the KO animals.

Finally, the analysis of the linker histone H1 revealed no alterations in total levels across the investigated cell types (Fig. S5E). Variant analysis, however, shows neuron‐specific changes in variant abundance and relative contributions (Fig. 4E). The analysis revealed opposing regulation of individual variants in granule layers, resulting in the observed unchanged levels of total H1 in those populations (Fig. 4E; Fig. S5E). Particularly, the DG and Cb KI brains are associated with a loss of replication‐independent variant H1.0 and gain of H1.4, while the H1 composition of pyramidal neurons seems largely unaffected by the mutation (Fig. 4E).

### Alteration of the global proteome

To obtain a comprehensive view of the functional consequences of Mecp2 loss‐induced histone dysregulation, we profiled the total cellular proteome across the investigated neuronal populations (Fig. 5A). The global proteome shows marked alteration in both mutants. As expected, Mecp2 is greatly reduced in mutant mice (Fig. S5F). To estimate the impact of Mecp2 on nuclear processes we focused on proteins with known nuclear functions for further analysis. Several hundred nuclear proteins are affected by the loss of Mecp2 (log_2_FC > 1) but show extensive region‐ and genotype‐specific alterations in chromatin‐ and RNA‐templated processes (Fig. 5B). In *Mecp2* ^*−/y*^ mice, the DG showed upregulation of proteins involved in DNA recombination, chromosome segregation, and RNA splicing, whereas CA neurons show a preferential increase in ribonucleoprotein complex assembly and learning‐related processes (Fig. 5C). Cb of the *Mecp2 null* mice was dominated by pathways linked to chromosome segregation and protein ubiquitination. In contrast, *Mecp2* ^*Y120D/y*^ animals exhibited upregulation of histone modifications, including methylation and lysine modifications, and widespread RNA splicing across all regions. Notably, transcription initiation and protein–DNA complex formation were significantly increased in the DG, whereas CA neurons exhibited enhanced nuclear transport and mRNA processing. Cerebellar changes in *Mecp2* ^*Y120D−y*^ mice were associated with vesicle and lysosomal pathways (Fig. 5C). These findings suggest that loss of Mecp2 function causes selective neuronal responses that converge on histone modification pathways, emphasizing epigenetic dysregulation as a key feature of RTT.

**Fig. 5:**
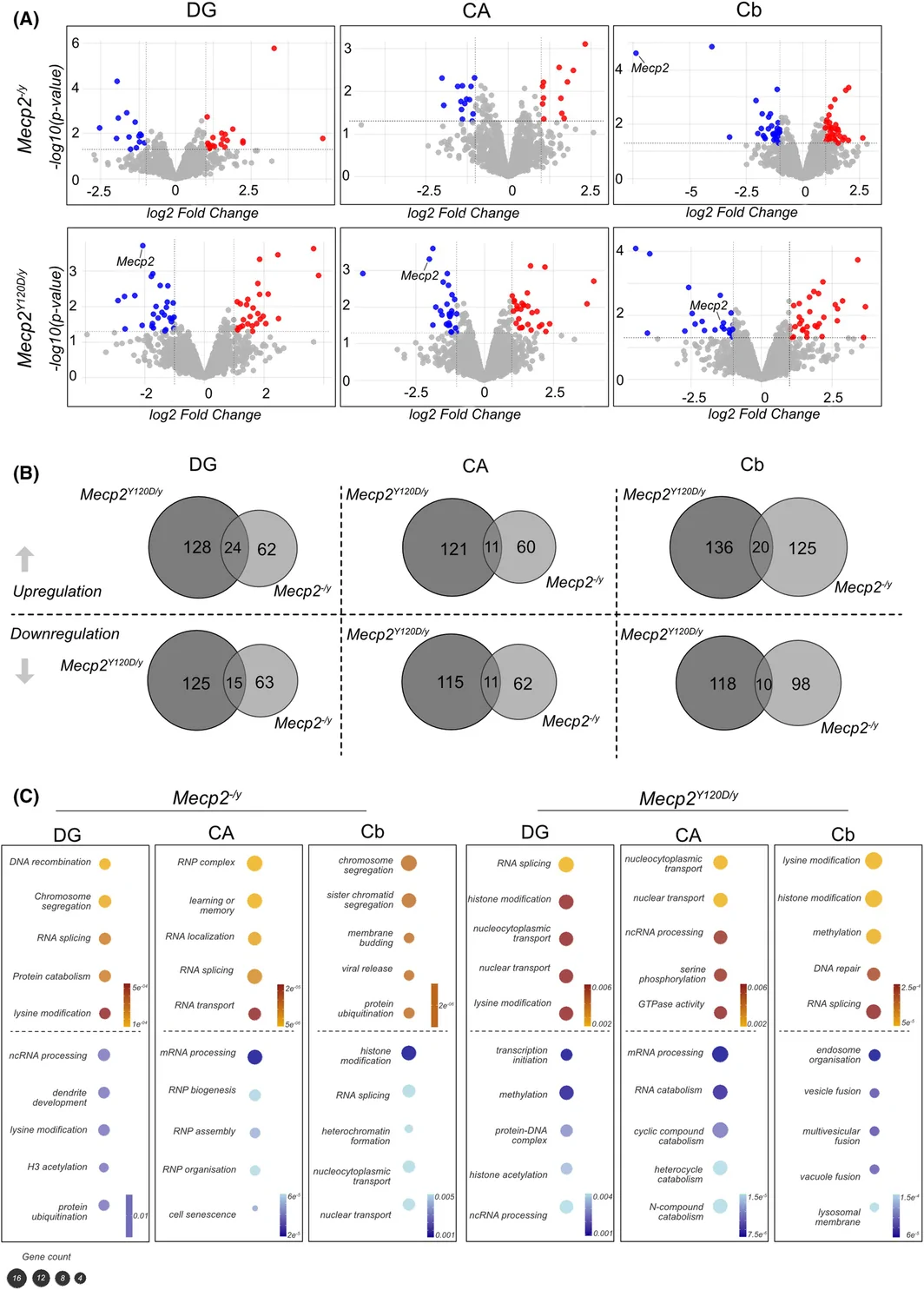
Loss of Mecp2 function induces global protein dysregulation. (A) Enrichment analysis of the global proteome between WT and *Mecp2* ^*−/y*^ *(top panel)* and *Mecp2* ^*Y120D/y*^ *(bottom panel)*. Highly enriched Mecp2 protein is indicated when detected in both groups. No Mecp2 was detected in *Mecp2* ^*−/y*^ DG and CA. Significantly altered proteins are highlighted (red = log_2_FC > 1, *P*‐value < 0.05; blue = log_2_FC > −1, *P*‐value <0.05). (B) Overlap of altered proteins between *Mecp2* ^*−/y*^ and *Mecp2* ^*Y120D/y*^. Upregulated proteins include all proteins with log_2_ fold change >1, downregulated proteins include all protein with log_2_ fold change < −1, when compared to WT. (C) Gene Ontology analysis of up‐ and downregulated nuclear proteins in *Mecp2* ^*−/y*^ and *Mecp2* ^*Y120D/y*^. Significance is color‐coded and gene count is indicated by dot‐size. The Top5 significantly altered GO terms are displayed. RNP, Ribonucleoprotein. *n* = 3.

## Discussion

Mecp2 has been shown to modulate chromatin structure and function on multiple scales, disruption of which is associated with RTT [1, 10, 70]. However, due to the high heterogeneity of utilized model systems, RTT mutations and applied methodologies, the observations are inconsistent and understanding remains incomplete. Our pipeline enabled spatial profiling of histone proteoforms *in situ*, which then guided targeted LC‐MS analyses of affected cell populations. This allowed us to quantify histone variants and modification states from a relatively low number of pure neuronal cells (30000 – 200000 cells) in healthy and RTT mice. The results revealed surprising heterogeneity in histone composition across neuronal subtypes that are differentially affected by Mecp2 mutations (Fig. 6A).

**Fig. 6:**
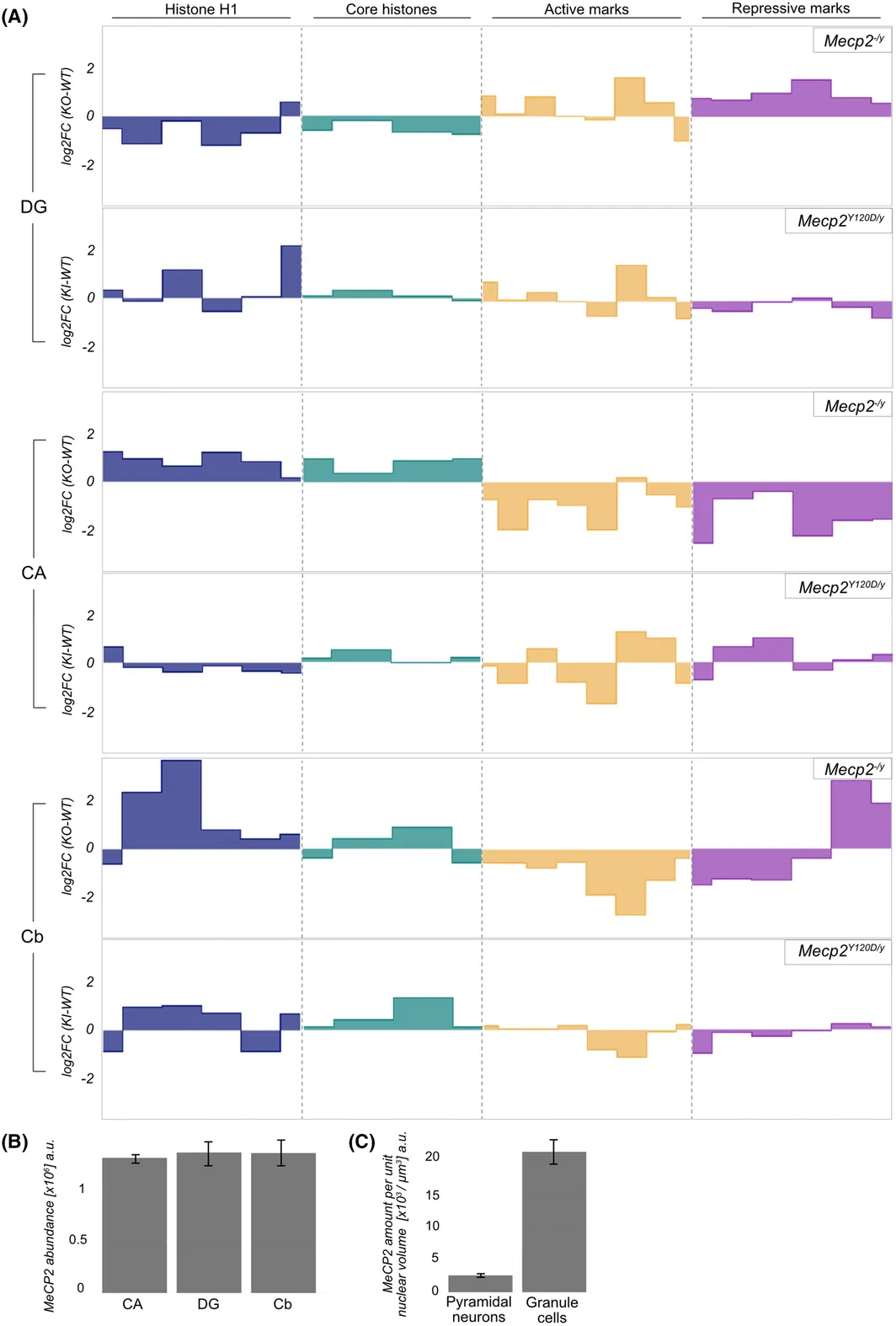
Global shifts in histone landscapes across Mecp2‐deficient neurons. (A) Area plots illustrating fold changes in linker histone H1 variants (blue), core histones (green) and active (orange)/repressive (pink) post‐translational histone modifications in DG, CA, and Cb neuronal populations of *Mecp2* ^*−/y*^ (top panels) and *Mecp2* ^*Y120D/y*^ (bottom panels) mice compared to WT. Histone proteoforms are ranked along the abscissa by name, each bar represents one proteoform. (B) Average abundance of *Mecp2* in WT CA, DG and Cb per nucleus. (C) Density of *Mecp2* per unit nuclear volume [μm^3^]. Error bars = SEM. *n* = 3.

Although we observe similar trends in histone proteoform alterations in both MSI and LC‐MS approaches, direct quantitative correspondence between MSI and LC‐MS signals is inherently limited. The complementarity of the two methods, however, underpins the strength of our approach. Signals detected in MALDI‐MSI represent composite or highly modified histone proteoforms that cannot be unambiguously resolved into individual isoforms, particularly for histones with minimal mass differences or extensive post‐translational modifications. As a result, MSI captures spatial shifts in dominant proteoform populations rather than the exact variant‐ or modification‐specific species, which then leads us to identification and quantification by LC‐MS.

Across neuronal populations, we observe significant variation in histone composition, suggesting localized alteration of epigenetic architecture that reflects a distinct response to functional loss of Mecp2 (Fig. 6A). As Mecp2 binding to chromatin has been shown to depend on the nuclear size and state of chromatin compaction [71], this neuron‐specificity may be connected to varying cellular Mecp2 concentration in neuronal subtypes (Fig. 6B,C). We not only detect changes in variant distribution but also fluctuations in total histone levels per cell, indicating either regional DNA copy number variation (DCV), a phenomenon documented in cortical neurons [72, 73], or possible existence of a stable, non‐DNA‐bound histone pool as the likely contributors [74, 75]. Nonetheless, our DNA‐based normalization of histone signals coupled with substantial involvement of unmodified histone forms as well as the lack of core histone stoichiometry (in Cb) support the latter. Comparing histone levels with publicly available RNA‐seq data shows family‐specific dysregulation in protein synthesis, suggesting that both histone production and/or degradation may contribute to this pool. However, the absence of relevant ChIP‐seq or related data in our cell populations of interest prevents us from further investigating this possibility [76]. Thus, it remains unclear whether these distinct chromatin compositions and varying histone levels have a specific regulatory function or represent downstream consequences of cellular shifts. The concordant changes we observe in the nuclear proteome in Mecp2 loss‐of‐function mutants support the hypothesis of chromatin‐wide functional disruption.

While the link between Mecp2 and core histones is still emerging, its association with linker histone H1 is better established. Matching previous reports, total H1 levels are increased in CA and Cb of Mecp2 *null*. However, the effect on H1 variants differs across neuronal populations and Mecp2 mutants, suggesting distinct H1 pool compositions with a variant‐ and cell‐type‐specific effect on chromatin compaction in RTT.

In addition to changes in isoform composition, we identified profound alterations in the global histone modification landscape across different neuronal populations, affecting both repressive and active marks. The global loss of histone modifications observed in CA and Cb of *Mecp2* ^*−/y*^ may hint at large‐scale disruption of chromatin domains. More ambiguous global effects in other neuronal populations indicate bidirectional chromatin shifts. These organizational disruptions could possibly arise from the varying local function of Mecp2 where it can induce destabilization of compacted chromatin as well as compaction of nucleosome‐free regions. Such context‐dependent alterations likely contribute to widespread transcriptional instability observed in RTT [16, 77, 78, 79].

Perturbation of chromatin states, particularly heterochromatin loss and global histone deacetylation, has been reported in multiple neurodegenerative disorders, autism spectrum disorders (ASD) and during normal aging in both mice and humans [80, 81, 82, 83, 84, 85, 86, 87]. Notably, most prior studies have investigated a limited set of canonical histone modifications rather than systematic profiling of the full landscape. Within these methodological restrictions, the patterns we observe are consistent with earlier reports linking large‐scale chromatin instability and impaired domain organization to cognitive impairment [86, 87].

Besides neuronal selectivity, histone proteoform changes exhibit a notable mutant specificity, which originates from distinct mechanisms leading to the loss of Mecp2 function. In knock‐in mice, the distinct alterations possibly stem from additive effects of disrupted binding of the MBD domain to 5mC/5hmC resulting in impaired recruitment of interactors [50] along with reduced protein levels due to increased degradation in mature neurons. In contrast, the Mecp2 *null* mice also include effects caused by loss of non‐MBD domains on chromatin, like the AT hook or C‐terminal domain, both of which have been implicated in chromatin binding [88, 89, 90, 91, 92]. Moreover, complete Mecp2 loss can trigger compensatory responses. Taken together, these effects likely account for the more pronounced chromatin alterations and phenotypic severity observed in the *Mecp2* ^*−/y*^ models.

Our findings underscore the need for spatially resolved analyses of RTT brains, given the intricate, cell‐type‐specific roles of Mecp2. While this study provides novel insights into the global landscape of intact histones and their modified proteoforms, it does not capture localized chromatin dynamics. Future efforts should aim to map these local effects across distinct chromatin compartments and neuronal populations, and to chart their progression over the disease course. Crucially, *Mecp2* ^*−/y*^ and *Mecp2* ^*Y120D/y*^ models display distinct epigenetic landscapes, reflecting mutation‐specific chromatin responses. These differences reinforce the potential importance of applying personalized disease management strategies tailored to the specific Mecp2 mutation involved.

## Materials and methods

### Animals

Both mouse lines were housed in the animal facility of the San Raffaele Scientific Institute of Milan. The *Mecp2* null strain was originally purchased from Jackson Laboratories (B6.129P2(C)‐Mecp2tm1.1Bird/J) and transferred on a CD1 genetic background [93]. The *Mecp2* ^*Y120D*^ mouse strain was generated as reported in Gandaglia *et al*., 2019 [50]. Both lines are maintained on a clean CD1 background by crossing *Mecp2* ^*−/+*^ or *Mecp2Y* ^*120D/+*^ heterozygous females with WT CD1 males purchased from Charles River Laboratories. Mouse genotype was determined by PCR as previously described [50, 93]. Mice were housed in groups of five in Tecniplast cages, on a 12 h light/dark cycle in a temperature‐controlled environment (21 ± 2 °C) with food and water provided *ad libitum*.

An initial symptomatic age at P42‐45 was chosen for tissue collection to ensure robust molecular phenotypes without secondary effects caused by a severe diseased state.

Male mice were used for analysis due to their consistent and reproducible phenotype in contrast to the more variable phenotype in female mice due to mosaic *Mecp2* expression.

Whole fresh brains were obtained from WT or mutant littermates sacrificed by rapid decapitation. As these mouse lines have been proven to show a stable phenotype, phenotypical scoring was not performed to avoid unnecessary manipulation of animals solely intended for tissue collection [50, 93, 94, 95, 96].

All procedures were performed in accordance with the European Community Council Directive 2010/63/UE for care and use of experimental animals (Reference number: n° 1217/2015‐PR); all the protocols were approved by the Italian Minister for Scientific Research and by the San Raffaele Scientific Institutional Animal Care and Use Committee in accordance with the Italian law.

### MALDI‐imaging mass spectrometry

#### Sample preparation

Three mouse brains of each genotype (WTs, *Mecp2* ^*−/y*^, *Mecp2* ^*Y120D/y*^) were halved mid‐sagitally and sectioned on a Leica CM3000 cryostat (Leica Biosystems, Wetzlar, Germany). 12 μm sections were thaw‐mounted onto indium tin oxide (ITO) slides (Bruker Daltonics GmbH, Bremen, Germany) coated with polyL‐Lysine (1: 1 in LC‐MS grade water, 0.1% NP40). Sections were stored in a sealed container at −80 °C until usage.

Sections were washed according to a pre‐established protocol to remove interfering salts and lipids [52]. In brief, proteins were precipitated *in situ* by incubating in 70% Ethanol and 100% Ethanol for 30s each, followed by 2 min incubation in Carnoy's fluid (60% EtOH, 30% chloroform, 10% acetic acid (v/v/v)). The slides were dried under vacuum for at least overnight at room temperature. Sections were then sprayed with matrix (10 mg mL^−1^ sinapinic acid (SA) in 60% ACN; 0.2% TFA) using a HTX TM Sprayer (HTX Imaging, HTX Technologies, Chapel Hill, NC, USA) with the standard manufacturer's method.

#### MALDI‐imaging mass spectrometry

Tissue measurements were carried out using a rapifleX MALDI tissuetyper MALDI‐TOF/TOF mass spectrometer (Bruker Daltonics GmbH). Samples were measured with 50 μm spatial resolution and within a mass range of 500–3200 m/z using positive reflector mode. The instrument was calibrated prior to measurement using a protein calibrant solution (Protein calibration standard I, Bruker Daltonics GmbH) spotted onto the same slide.

#### Histology and nuclear density determination

After MSI measurements, the matrix was removed and the tissue was stained with hematoxylin and eosin (HE). Nuclear density was determined as previously described [52]. In brief, slides were scanned with a Mirax Desk digital slide scanner (Carl Zeiss Microscopy GmbH, Jena, Germany) at 20× objective magnification. Images of HE‐stained slides were analyzed using Definiens TissueStudio3 (Definiens AG, Munich, Germany). Nuclei were identified and quantified based on morphology, size, pattern, and neighborhood. The generated HE images were also used for co‐registration to MSI data to allow for histological correlation.

#### Data analysis

MSI data were normalized to the total ion count (TIC) using fleXImaging (Bruker Daltonics GmbH). The MSI data files were imported into SCiLS Lab 2016b (Bruker Daltonics) and unbiased hierarchical clustering was performed using the segmentation pipeline with a minimal m/z interval of 5 Da [97]. Regions of interest (ROIs) were selected based on a combination of the outcome of the clustering results, literary knowledge of RTT and normal brain physiology and correlation with morphological staining. Neuronal populations of DG, CA, and cerebellar granule cells (Cb) were analyzed. Regional mass lists were exported and peak picking was carried out in mMass v 5.5.0 [98] and masses were matched to theoretical histone masses.

### LC–MS/MS

#### Laser capture microdissection

Three mouse brains were cryosectioned for *Mecp2* ^*−/y*^, *Mecp2* ^*Y120D/y*^ and their respective WT littermates. Due to limited availability, two replicates were used for Mecp2^Y120D/y^ WT littermates. 50 μm thick sagittal sections were produced and mounted onto metal‐frame PET slides (Leica Biosystems). The sections were stained with Hematoxylin and Eosin (HE) staining to visualize brain regions for LCM. A Leica LMD 7000 with a 5× objective was used for LCM of brain regions. The laser setup was configured as follows: Pulse frequency—120, maximum pulse energy—50, aperture—17, Speed—10, Head current—100 %, Offset—65. Hippocampal neuronal layers dentate gyrus granule cells (DG) and cornu ammonis pyramidal cells (CA) as well as the cerebellar granule cell layer (Cb) were dissected from each brain. Excisions of ten serial sections were pooled for each biological replicate and sectioned areas traced for post‐measurement normalization. Separate sample pools of ten serial sections for each biological replicate were prepared for total proteome, histone, and DNA extraction individually.

#### Acidic extraction of histones

Laser‐microdissected samples were processed using an in‐house protocol for histone extraction based on previous work [53]. In brief, lysis buffer (60 mm KCl, 15 mm NaCL, 4 mm MgCl_2_, 15 mm HEPES pH = 7.6, 0.5% Triton‐X, 1 mm DTT, 1 tablet of Roche complete protease inhibitor) was added to the samples and sections were manually homogenized using Micro‐homogenizers, PP (Carl Roth GmbH+Co. KG) to enhance tissue lysis. The samples were centrifuged and the resulting pellet containing the nuclei resuspended in 0.2 m H_2_SO_4_ and incubated overnight at 4°C to ensure complete histone extraction. Basic proteins were precipitated with trichloroacetic acid (TCA) and pelleted through centrifugation. The pellet was washed multiple times with ice‐cold acetone to remove any remaining acid.

Lysine residues were propionylated using NHS‐propionate prior to tryptic digestion. Each sample was incubated with 20 μL of 34.2 mg·mL^−1^ NHS‐propionate in ACN for 30 min at 50°C. NHS‐propionate was chosen over propionic anhydride due to its higher specificity and efficiency at lysine (K) residues [99]. Fixed amounts of stable isotope labeled (SIL) histone peptides, both modified and unmodified (label: R(15N; 13C); 500 fmol·μL^−1^; JPT Peptide Technologies GmbH, Germany) were added to the reaction mix prior to digestion to serve as internal control for identification and monitor digestion efficiency [100, 101]. Samples were digested at 37 °C overnight with trypsin (Sequencing‐grade Modified Trypsin, Promega GmbH, Madison, WI, USA), with detection of SIL histone peptides as a monitor for protein digestion. Tryptic peptides were desalted and purified using C18 Stagetips. The samples were then dried in a vacuum desiccator and resuspended in 10 μL of 0.3% TFA and 2% ACN for LC‐MS/MS measurement.

Extraction efficiency between groups was estimated by calculation of the proportions of affected/unaffected proteins. Proteins were defined as affected by Mecp2 loss‐of‐function when log_2_ fold change > 0.5. Further, cell‐type‐specific basic non‐histone proteins were defined as control proteins. Control proteins were defined according to the following criteria: (a) log_2_ fold change < 0.07. (b) Stable expression across all groups.

Sets of control proteins were defined in a cell‐type‐specific manner, as house‐keeping genes cannot be assumed for stable expression across all cell types [102].

#### DNA purification and measurement

Genomic DNA was purified from the designated pool of 10 laser‐microdissected sagittal sections per animal using the NucleoSpin Tissue Kit (MachereyNagel GmbH&Co. KG, Düren, Germany). To enhance tissue lysis, tissues were initially homogenized with Micro‐homogenizers, PP (Carl Roth GmbH+Co. KG). DNA concentration was determined using the Qubit dsDNA High Sensitivity Assay Kit (Thermo Fisher Scientific Inc., Waltham, MA, USA).

#### Proteomics sample preparation

Laser capture microdissected tissue was processed using the PreOmics iST Kit for mammalian tissue according to the manufacturers guide (PreOmics GmbH). Tissue lysis was enhanced by manual homogenization with Micro‐homogenizers PP (Carl Roth GmbH+Co. KG) after the addition of lysis buffer. Protein digestion with trypsin/LysC was carried out for 3h at 37 °C. Resulting peptides were desalted, purified and dried in a speedvac, followed by resuspension in 12 μL “LC‐LOAD” for LC‐MS/MS measurement.

### LC–MS/MS

For LC–MS/MS, the peptides were injected into an Ultimate 3000 RSLCnano system and separated either in a 25‐cm analytical column (75 μm ID, 1.6 μm C18, Aurora‐IonOpticks) with a 50‐min gradient from 2% to 35% acetonitrile in 0.1% formic acid. The effluent from the HPLC was electrosprayed directly either into an Orbitrap QExactive‐HF (Proteome analysis) or an Orbitrap Exploris 480 (Histone extraction) instrument both operating in data‐dependent mode to automatically transition between full‐scan mass spectrometry and MS/MS acquisition. Typical mass spectrometric conditions for both instruments were: spray voltage, 1.5 kV; no sheath and auxiliary gas flow; heated capillary temperature, 275 °C; ion selection threshold, 5 × 10^3^ counts; dynamic exclusion, 20 s. Specific settings are described below. QExactive‐HF settings: Survey full‐scan MS spectra (from m/z 375–1600) were acquired with resolution R = 60 000 at m/z 400 (max IT 60 ms, AGC target of 3 × 10^6^). The 10 most intense peptide ions with charge states between 2 and 5 were sequentially isolated to a target value of 1 × 10^5^, fragmented at 27% normalized collision energy and acquired with resolution 15 000 at m/z 400 (max IT 60 ms). Exploris 480 settings: Survey full‐scan MS spectra (m/z 250–1200) were acquired with resolution 60 000 at m/z 400 (max IT 40 ms, AGC target of 3 × 10^6^). The 15 most intense peptide ions with charge states between 2 and 6 were sequentially isolated to a target value of 2 × 10^5^, fragmented at 30% normalized collision energy and acquired with resolution 15 000 at m/z 400 (max IT 40 ms).

### Data analysis

#### Protein identification

MaxQuant 2.0.1.0. was employed for protein identification [101]. Default MaxQuant conditions were used with parent and fragment ion mass tolerances of 4.5 and 20 ppm respectively. Allowance for missed cleavages was set to 2. For database searches, the reviewed mouse canonical protein database from Uniprot was used. Additionally, the following conditions were used: protein FDR and peptide FDR of 0.05 and 0.01, respectively; minimum peptide length of 5; variable modifications of oxidation (M), acetyl (protein N‐term), acetyl (K), methyl(KR), dimethyl (KR) and trimethyl (KR); fixed modification of carbamidomethyl (C); peptides for protein quantification, razor and unique; minimum peptides of 2; minimum ratio count of 2. Protein identification was carried out based on a unique peptide detected in all or at least 50% of the replicates.

#### Histone analysis

Due to the high sequence similarity between histone family members, quantification was carried out on the peptide level to ensure accurate quantitation. For each member of the histone family (H1, H2A, H2B, H3, and H4), one shared peptide across their respective isoforms and variants was used to approximate total histone abundance for that respective member. Peptides were chosen based on the following criteria: (a) maximum completeness of detection across samples, (b) identification by MS/MS and (c) maximum coverage ensured by peptide length. Likewise, individual histone isoforms and variants were quantified based on a unique peptide that was chosen based on using the same criteria as above. As H1.0 does not share any peptides with the remaining H1 variants H1.1‐1.5, a separate peptide was used for H1.0. Further, intensities from all the unique H1 variant peptides were summed up to estimate the total H1 pool. Peptide sequences can be found in Files S1 and S2, as well as Table S5. Peptide intensities were normalized to the total DNA concentration of the respective regions (from the serial sections from the same brain), sample batch corrected using ComBat [62] and imputed by ‘tiny’ imputation in Perseus 2.0.6.0 [101] where necessary.

Detection of post‐translational modifications of core histones H3 and H4 peptides were validated manually on the MS1 level using Skyline [103]. Peak assignments were supported by stable‐isotope labeled (SIL) histone peptides and fragment spectra, where needed. Precise identification was further guided by spike‐in internal standards (SIL peptides). Modified peptides had to be present across samples to be considered for analysis. Peptide abundance changes were visualized both as relative (within genotype) and absolute changes (across genotypes). The relative abundance of each modified peptide was calculated as percentage of the overall peptide according to previous work [60], to visualize contribution of unmodified and differentially modified peptides to the total peptide abundance. For fold change comparison of absolute values of modified peptides between WT and Mecp2 deficient brain cells, modified H3 peptides were normalized to unmodified H3_41‐49 peptide and modified H4 peptides were normalized to unmodified H4_46‐57 peptide. Intensities were further normalized to DNA concentration and batch corrected using ComBat [104].

Statistical significance was estimated using LIMMA statistical testing, which uses the full dataset to reduce the predicted sample variances for each protein to a pooled value. This approach produces significantly more stable and strong inference compared to traditional t statistics, especially when the number of replicates is minimal (Table S6). The propionylation reaction efficiency was assessed by analyzing propionylation of the histone peptides using ProteomeDiscoverer (Thermo Fisher Scientific Inc.). Histone sequences were in‐silico digested with a maximum of 2 missed cleavages, a precursor mass tolerance of 10 ppm, a fragment mass tolerance of 0.02 Da, 5 max. equal modifications per peptide, 4 max. dynamic modifications per peptide, propionyl (+56.026 Da) as N‐terminal modification and dynamic modification on K, S, T with a max. Delta Cn of 0.05. Efficient propionylation of lysine (K) residues without significant propionylation at other possible amino acid residues was observed (File S3).

Whole proteome data were filtered for nuclear annotated proteins (Uniprot) to generate the nuclear proteome. Enrichment analysis was carried out in Imshot [105] using LIMMA statistical testing. Gene Ontology (GO) analysis was also performed in Imshot, using the global mouse proteome as background and Benjamini–Hochberg correction.

### Total RNA‐seq data

Publicly available total RNA‐seq data were used for independent validation of our proteomics findings. Datasets were identified by manual curation and the Mecp2pedia resource [49, 62, 63, 64, 65, 66, 67]. RNA‐seq information of the histone gene products was extracted from the specific brain cell populations of interest (DG, CA, and Cb) for RTT mice (*Mecp2* ^*−/y*^). The corresponding fold changes were plotted in quantification plots and analyzed for correlation with the histone protein levels under similar conditions (Fig. S2C).

## Conflict of interest

The authors declare no competing interests.

## Author contributions

Planned experiments: FS, AI and SL, Performed experiments: FS, GR and SL, Analyzed data: FS, IF and SL, Contributed reagents or other essential material: MVA, IF, NL, AI and SL, Writing–original draft: FS, AI and SL, Writing–review & editing: FS, GR, MVA, IF, NL, AI and SL.

## Supporting information


**Fig. S1.** Histone proteoforms are sufficient to separate regions of interest in RTT brains.
**Fig. S2.** Exclusion of confounding factors influencing histone abundances.
**Fig. S3.** Alteration of the H3 pool, H2A/B isoform coverage maps and comparison of histone abundances with respective mRNA.
**Fig. S4.** Alteration of specific histone modifications and corresponding writers/erasers in *Mecp2* ^*−/y*^ along with H1 variant coverage maps.
**Fig. S5.** Alteration of histone proteoforms (including linker histone H1) in Mecp2Y120D/y and quantification of Mecp2.
**Table S1.** MSI results. Masses contributing prominently to the loadings plot in Fig. S1A (highlighted) are listed.
**Table S2.** MSI results. Detected masses used to plot the heatmap in Fig. 1D that were assigned to theoretical histone masses based on previous work.
**Table S3.** Information about compaction ability scoring used for Fig. 3C (Sheets: Scoring system, DG, CA, Cb and compaction). 1 = weakest effect on chromatin compaction; 6 = highest effect on chromatin compaction. Based on previous work.
**Table S4.** Information about compaction ability scoring used for Fig. 3. Compaction scoring in DG, CA, Cb.
**Table S5.** Histone coverage maps for all histone isoforms. Covered sequences are highlighted.
**Table S6.** Results of LIMMA statistical analysis between WT and mutant groups.

**File S1.** LCM LC–MS/MS results for histone quantification containing peptide name, sequence and intensities. Normalized and log‐transformed intensities were used as input for quantification of total, isoform and variant histone abundances based on MaxQuant output ‘peptides.txt’.

**File S2.** LCM LC–MS/MS results for quantification of histone post‐translational modifications. Normalized and log_2_‐tranformed intensities were used as input for absolute quantification (Sheet: log_2_Intensities). Relative levels of a modified peptide were calculated as percentage of the overall peptide (Sheet: Percentages).

**File S3.** Propionylation status of H3 and H4 peptides.

## Data Availability

The mass spectrometry proteomics data have been deposited to the ProteomeXchange Consortium via the PRIDE [106] partner repository with the dataset identifier PXD065165.
