# Supplementary material for: Spatially resolved mapping of histones reveals selective neuronal response in Rett syndrome
Source: FEBS J. 2026 Apr 1;293(19):5814–36. doi: 10.1111/febs.70534 (PMC13628532; doi:10.1111/febs.70534)
Supplement: Supplementary file 1 [file FEBS-293-5814-s001.pdf]

Peptide	H3 9 17 unr	H3K9me1	H3K9me2	H3K9me3	H3K9ac	H3 18 26 ur	H3K18ac	H3K23ac	H3 27 40 ur	H3K27me1	H3K27me2	H3K27me3	H3K36me1	H3K36me2	H3K27ac	H3.1K36ac	H3 54 63 ur	H3K56ac	H3K56me1	H3 73 83 ur	H3K79me1	H3K79me2	H3 117 128	H3K122ac	H4_4-17_4p	H4 4-17 3p1	H4 4-17 2p2	H4 K20 unm	H4K20me1	H4K20me2	H4K20me3
KI1.CA	11.1344929	9.42912483	10.0503991	9.06254005	10.2583447	16.0674438	12.6902523	14.5755157	11.2091053	9.76897272	13.5425485	12.9524645	9.28041563	12.6528112	11.4719664	8.46993828	17.3951321	11.6902409	12.3333454	18.7025986	14.9547405	14.2532234	18.1813221	12.9611549	14.8549938	15.2288895	12.7777481	6.28210592	9.90049934	11.0445404	9.5253582
KI2.CA	14.4538212	12.7654638	10.8749177	12.546195	14.04076	19.2438755	15.016427	17.4448586								12.9486027	21.3569317	14.7616072	18.1201191	22.5913582	19.2705746	17.7516727	22.5133801	17.2167034	18.8613224	18.3706245	15.0728416	9.26951218	11.3111572	13.8802891	12.2894707
KI3.CA	12.1209736	10.8780079	10.3731756	9.12271309	10.7425461	16.2562523	13.0639591	14.5996323	10.773934	9.19556165	11.7154044	11.1832816	9.52253608	11.7402973	9.21845798	2.77098727	15.214386	10.9156799	10.0070915	16.5807724	13.3426914	12.2453289	16.2743187	10.2454987	14.25214	14.4832125	11.6766138	7.5874629	9.65130424	11.8153267	10.2096395
KO1.CA	11.7994642	11.0281935	11.0762845	9.57840824	11.323184	18.1781998	13.4075804	15.7602129	12.7452231	10.2124608	13.3336745	13.1483296	11.5914876	13.6561249	13.9275372	9.90641689	18.0198021	15.3829145	15.3666029	19.3826408	16.1261387	14.4013109	19.4002018	14.6769552	16.7849731	17.3252983	14.8946342	9.02448082	12.2892361	12.5641956	10.9124889
KO2.CA	10.7591047	11.1483984	11.0649487	5.42733288	10.2093019	16.549778	13.2214918	14.8819227	10.8936475	10.2298598	11.9097126	11.9626971	9.55663922	11.8570784	10.2379184	2.42947078	15.8965292	9.71342754	9.72432041	17.0934677	13.7437239	12.3389139	16.962534	8.73353291	13.992589	14.3289289	11.9152384	5.21162081	9.23523331	10.6191549	9.1722517
WTI1.CA	11.3475676	10.4498587	10.0180091	9.20483494	10.4385805	17.5824966	14.3323202	14.9978094	11.8233714	11.1396283	12.8919971	12.4815334	10.3653162	12.9620141	11.7075482	8.32571507	16.9542885	11.4663401	13.0907335	18.2139187	15.3452368	13.6630239	18.4730587	12.3195162	15.993783	16.1917801	13.6367922	6.71918058	10.3926325	11.9108934	10.1564608
WTI2.CA	11.3471766	9.75179768	9.8869529	9.33957005	10.4171534	17.6877384	13.0897989	14.9477949	11.6827014	10.3298164	13.7757402	12.3028954	9.985272	13.0904388	12.2628554	9.43705463	17.2631435	13.3472147	13.715766	18.6125755	15.3381872	13.8508234	18.5984135	13.0876856	15.5592976	15.9093018	13.6260986	7.70632553	11.4799538	11.9778481	9.94960022
WTO1.CA	12.9039307	12.8651505	11.6214171	10.483695	11.8648548	18.0346088	14.9656305	16.309597	12.2478261	12.1612128	13.8797796	13.6352172	11.7016278	13.4844657	13.6046258	8.17298317	17.6775837	12.7704992	15.145195	19.0005341	15.6557951	14.2038794	18.9412098	14.3405161	16.7190628	16.7543125	14.0496082	6.43462753	11.4295712	13.4748421	11.6802044
WTO2.CA	12.0398178	11.654026	10.4211888	9.80834579	11.5727596	18.05513	14.9776878	16.4591084	12.0233404	10.9532723	13.4304173	12.6032758	9.98623711	13.4136691	13.1746479	7.23912048	17.7887154	14.8785343	15.2804794	19.1722603	15.7924576	14.6885281	19.3240452	14.4408855	16.5421524	16.8876781	14.4886456	9.47736168	12.1022778	13.5519104	12.2874908
WTO3.CA	11.4640598	9.93990326	9.72003	9.57898712	10.9628162	17.7610474	15.7089844	15.2630253	11.736156	10.3581543	12.5797147	12.5864199	9.47313119	12.8797841	12.2491131	8.82472229	17.099165	12.538393	13.6859016	18.4716301	15.0439529	14.1822376	18.4930229	13.0835724	15.637785	16.0133686	13.8271027	7.74685287	11.2614021	12.3663177	10.6393623
KI1.DG	10.0942974	10.4356518	9.5149904	8.60115814	9.41880608	15.9624252	12.2940321	14.5422478	10.5795045	8.14058236	11.2763447	10.8094095	9.17975419	11.5217082	9.80360943	1.6843735	15.1764603	8.53365707	8.12979603	16.5182457	13.1008234	12.1406145	16.419014	8.31313324	14.0831089	14.5416546	11.5592537	4.56932211	9.00618362	10.5202465	9.1348381
KI2.DG	10.9185896	10.1165075	8.29240133	8.87317467	10.4042578	17.398922	15.6364441	15.1083937	10.5298234	7.14401348	12.1241196	12.3590725	8.3360072	12.3039823	12.0393907	8.99106503	17.6543083	14.7714357	13.4318771	19.1696758	15.1411657	15.1754417	18.4191418	12.5378237	15.017951	15.7859697	13.6680651	6.97538519	10.3073225	12.8863001	11.2869101
KI3.DG	11.454133	10.0552988	11.0808285	9.85016537	10.5293474	17.5090752	15.4598837	15.2563143	11.5049506	9.47362545	12.424991	11.7291196	9.88178037	12.6398797	10.5280479	7.83270407	17.2389698	11.7081614	11.896018	18.4334755	14.8552446	13.8380766	18.1630745	11.4286776	16.0316105	16.8146706	13.9066019	6.69832134	10.9448595	12.4603186	10.9942846
KO1.DG	12.2864771	11.7321997	13.6191438	10.2726736	11.4271498	17.0980129	13.7357798	15.8472815	12.0103698	10.6693779	12.7777823	11.5477392	10.4584083	12.9143036	10.1059671	6.79098225	16.8208351	11.3340521	12.065526	18.0830002	14.5779905	12.6304998	18.0370636	10.985465	15.5442591	16.3336182	13.2853727	8.68003368	10.6326933	13.033721	11.3693686
KO2.DG	11.0590601	10.8799353	12.4883908	9.23974705	10.310813	16.3138504	12.8289289	15.0414705	10.8332409	8.86234398	11.520584	11.9739437	9.23032214	12.1266311	9.38358942	4.45730448	15.9102468	12.0310698	9.85236835	17.1398754	13.7922316	12.6995287	17.1463966	8.46983624	14.2231417	15.0229006	12.1508589	6.08918619	9.33000469	11.8815985	9.93906498
WTI1.DG	10.8714304	11.3228121	10.8909078	9.2339201	9.76110172	16.6430569	13.1311235	15.0474691	11.0642502	9.47730368	11.9417816	11.5749722	9.54402602	12.29629	9.64185735	3.68226886	16.1244583	10.1003485	10.1073256	17.4428291	14.0691748	12.2559042	17.5271626	8.69680119	14.7966242	15.6841879	12.6450548	5.6491828	9.863801	11.9830103	9.90006161
WTI2.DG	11.1174631	10.014678	9.36439776	9.80858517	10.3724661	17.768734	12.8938665	14.8921709	11.6648393	8.55923018	13.3093956	12.1731021	10.0845078	13.1886642	11.2090635	8.33559704	17.5888386	14.3175611	12.2831812	18.7942886	15.0093584	13.6317625	18.3214264	11.5460443	15.9077263	16.514616	13.7764263	6.35222673	11.358139	12.5165253	10.8131371
WTO1.DG	11.1801567	9.67493248	10.9705876	9.529459	10.5072136	16.7352238	13.4996281	15.6311111	10.9978427	9.02286356	11.6812947	11.1138965	8.96901	12.3936458	10.4414146	5.33661413	16.8432217	9.69252205	10.5930424	18.1764622	14.3216324	13.5670166	17.71241	10.3512688	14.4586496	15.4988985	12.7601004	6.40159702	9.77398586	12.0971212	10.4106054
WTO2.DG	9.96007729	8.1837616	7.53255243	8.65608406	9.77360725	16.1150455	14.2237968	14.3216248	9.59693233	6.78584411	11.1993081	9.6384249	6.74765516	11.2709952	8.4938313	5.06733513	16.4041386	6.59896469	6.49508476	17.7230301	13.3770924	13.6068211	16.7263851	9.59918594	12.0804892	13.4450655	11.0102816	3.85860109	7.65185213	11.3144903	9.70226574
WTO3.DG	10.5282393	8.98791218	9.38707411	8.86557674	9.83503628	16.8131237	11.8751192	14.0440149	11.0218115	8.17235139	11.5564693	11.6935514	9.16580707	12.2681987	10.2855299	6.87613773	16.6621304	10.7504969	11.3695459	18.0854359	14.3720045	13.773818	17.8963413	11.4227304	15.2333488	15.929945	13.2455215	5.51802444	10.3765354	11.6493549	10.2062321
KI1.Cb	9.2022171	8.50735474	8.53134576	7.61978769	8.3532238	14.9983482	10.8768673	13.5919456	10.7019547	9.86865307	10.725822	9.88873522	10.1472284	10.1762218	9.97654351	6.08579874	14.2949734	5.09130907	4.57071161	15.6054745	12.4371891	10.7323666	15.650301	6.76511812	13.9127455	14.4921713	10.6702108	1.68325102	8.26748753	9.58664799	7.39795351
KI2.Cb	9.34970951	8.87618542	8.29209685	7.7087779	8.77195454	15.4097109	11.2716589	14.0915785	11.0122241	9.77210145	10.8356815	10.1836459	10.2046517	10.5579471	9.93216472	4.14076757	14.0485344	5.46761465	5.24106741	14.7294054	12.6918955	11.5796289	15.772747	6.68650007	14.0234919	14.7833719	11.1370382	1.58562148	8.27903652	9.90418911	7.58113718
KI3.Cb	8.95642757	8.06657505	7.1316139	7.33642244	8.16094112	14.9544878	10.8971777	13.5772753	10.5124069	9.5352488	10.467808	9.61399951	9.45382587	10.0316241	9.42229538	3.15864229	14.3316545	5.1049962	3.84734988	15.3935881	12.0448561	10.9684782	14.6028032	4.65917444	13.5798969	14.292202	10.4293766	2.21344376	7.80920219	9.26719284	7.00046921
KO1.Cb	12.9315548	10.7499352	11.6827204	11.0637722	13.2789478	17.1631374	13.781292	16.6648598	12.2728649	10.7958309	12.9883692	11.545421	8.97250023	12.2672518	12.2027989	5.53042364	17.4036427	9.03159332	10.8303699	18.1767712	13.57831	13.3534985	18.197691	10.7922935	14.4240122	15.817935	13.0108385	7.62273359	10.228694	13.7490835	13.6565409
KO2.Cb	9.51792145	9.4077549	7.40937724	7.45018911	8.49097633	14.3771057	10.3778591	12.9558125	10.3755801	9.41913847	9.9691487	8.927382	9.00706133	9.68468108	9.26156856	2.11436629	12.8164616	4.10717726	2.54950881	14.6099396	11.3406754	9.86481476	14.7186317	5.66096449	12.9429636	13.5262861	9.87252331	2.6601572	7.58338261	9.6044054	7.19606876
WTI1.Cb	9.42186737	8.86053658	11.0101877	7.69087219	8.55498123	15.8934116	10.6778355	13.9271755	11.5278606	10.2382753	11.158382	10.487223	10.9840119	10.7950878	9.73997943	8.65310097	15.2162075	6.23771095	6.33723259	16.5014439	13.2527046	10.7536716	16.4234257	7.71841621	12.791955	15.3959799	11.8268576	2.09858632	9.05380726	9.79737759	7.44419384
WTI2.Cb	9.30712605	8.65103626	6.28519524	7.2118597	8.20714283	15.1172266	10.9946585	13.7170801	10.9393805	9.82244552	10.3100271	9.85171247	10.4747408	10.2508379	9.72763122	2.4453547	14.360425	4.84707594	4.68596029	14.7772608	12.3696756	11.0421705	15.4554243	6.5144515	14.0586958	14.5107336	10.7465773	1.29439557	8.47368336	8.90434742	7.46028614
WTO1.Cb	9.08853149	8.34479332	8.02532798	7.46644974	8.34045982	15.197401	11.0971489	13.6220455	11.0413999	9.81523183	10.7612166	9.88332787	10.3101575	10.3135318	9.81015591	6.4293232	14.1199589	5.38274574	3.50881791	15.7123604	12.5889359	10.4730053	15.5539913	6.54691696	13.7256927	14.5141602	10.8373642	1.1964494	8.21801758	9.2751255	7.03295898
WTO2.Cb	10.4851761	9.39678383	8.74528754	9.22325134	10.211731	16.855669	12.8398933	15.5877476	12.307765	10.8897159	12.6773366	11.2465921	10.4797077	12.1286062	10.6417504	3.86801934	16.6293259	6.7425828	9.05919075	17.5152359	13.6685429	12.0275669	17.587656	8.5266037	13.8407221	15.531785	12.2611456	4.14821863	9.55340099	9.35914612	9.26601315
WTO3.Cb	15.3461666	14.440897	15.4881101	15.4708691	15.6445694	18.6744289	13.865818	17.4117966	14.8974658	14.8618721	14.6495339	13.3744121	14.0311087	14.1475075	13.2363149	6.5813446	17.2200584	8.04400826	6.8822319	18.9108181	15.9751701	13.3251057	19.100359	10.0022011	13.9751816	14.1780834	10.6936226	3.72149682	9.03861809	8.22005844	9.54437637

Percentages

Peptide	H3_9_17	H3K9me1	H3K9me2	H3K9me3	H3K9ac	H3_18_26	H3K18ac	H3K23ac	H3_27_40	H3K27me1	H3K27me2	H3K27me3	H3K36me1	H3K36me2	H3K27ac	H3K36ac	H3K27K36aca	H3_54_63	H3K56ac	H3K56me1	H3_73_83	31 H3K79me	31 H3K79me	H3_117_128	H3K122ac	H4_K18-23	H4K20me1	H4K20me2	H4K20me3
KI1.CA	39.0474168	11.9736173	18.4182152	9.28693401	21.2738167	68.8810103	6.62923711	24.4897526	6.54681455	2.41272008	32.996391	21.9196423	1.71963629	17.8085606	7.8552337	0.98052486	7.76047659	95.3187598	1.82740584	2.8538344	89.2691058	6.64482416	4.08607	97.3873827	2.61261729	2.00432225	24.6157096	54.4005181	18.97945
KI2.CA	41.4675646	12.8665648	3.4701749	11.0523881	31.1433077	74.5848765	3.98164255	21.433481	1.41412248	6.32380636	18.3950444	3.86913372	2.97832656	0.26168625	25.570124	1.89094918	39.2968071	89.5720737	0.92636557	9.50156073	88.1055563	8.81754775	3.07689595	97.5189781	2.48102189	2.6552039	10.9318655	64.8753969	21.5375337
KI3.CA	44.8420879	18.9459273	13.3519988	5.6120235	17.2479625	70.0975659	7.66879165	22.2336425	12.3228887	4.12643637	23.6659183	16.3658458	5.17611876	24.0778048	4.19244741	0.04803807	10.0245018	92.7748068	4.71401102	2.51118222	86.5412872	9.17199581	4.286717	98.4915076	1.50849236	3.32498193	13.9016803	62.3021399	20.4711979
KO1.CA	32.0002558	18.7489875	19.3845038	6.86355276	23.0027002	81.715884	2.99368886	15.2904272	9.14164556	1.57974487	13.7456704	12.0884759	4.1088037	17.188309	20.7460974	1.27778731	20.1234658	75.7724042	12.1822787	12.0453171	88.0052071	9.2088106	2.78598233	96.3522693	3.64773067	3.85472657	37.0495413	44.8284696	14.2672625
KO2.CA	23.5081865	30.7898943	29.0594512	0.5837083	16.0587597	70.7073137	7.03963399	22.2530523	11.0794166	6.99353774	22.4069616	23.2451784	4.38568405	21.6042147	7.03271083	0.03137227	3.2209239	97.3113332	1.33925834	1.34940849	88.0951754	8.64128767	3.26353691	99.6678162	0.33218384	1.32839818	21.6051093	56.384288	20.6822045
WTI1.CA	37.124517	19.9261552	14.7714725	8.40686398	19.7709913	78.6288483	8.26383438	13.1073174	9.74113964	6.06432655	20.4314007	15.3722085	3.54561366	21.4474349	8.98966493	0.86240306	13.545808	91.6605334	2.04242247	6.29704412	84.7763575	11.6068865	3.61675605	98.6147087	1.3852913	1.63560933	20.86887	59.7779506	17.7175701
WTI2.CA	40.5201593	13.4095514	14.7265172	10.0767705	21.2670017	83.964175	3.46717716	12.5686478	7.03786308	2.75538229	30.0267454	10.8177719	2.17001957	18.6729101	10.5216677	1.48399958	16.5136404	86.8219051	5.7519749	7.42612003	87.7030475	9.06411272	3.23283973	97.853738	2.14626203	2.58234371	35.3174499	49.8735695	12.2266369
WTO1.CA	32.7008835	31.8335798	13.4426407	6.10936751	15.9135285	70.340295	8.38204417	21.2776609	5.96485648	5.61728891	18.4870099	15.6043672	4.08486699	14.0561254	15.2769668	0.3539564	20.554562	82.9062879	2.76317342	14.3305387	88.1514296	8.67686603	3.17170439	96.0416596	3.95834039	0.49396299	15.7515126	65.0144899	18.7400345
WTO2.CA	33.0318274	25.2812011	10.7566419	7.03384168	23.8964879	69.0009718	8.17434201	22.8246862	6.99878685	3.33349782	18.5607329	10.4616627	1.70527162	18.3465071	15.5453624	0.25399728	24.7941813	76.4058691	10.1642405	13.4298904	87.6606147	8.42136553	3.91801974	96.7224468	3.2775532	3.22263957	19.8788021	54.2966605	22.6018978
WTO3.CA	38.1178183	13.2529082	11.3794879	10.3196414	26.9301442	70.5139929	17.003675	12.4823321	9.29531974	3.57638667	16.6801824	16.7578873	1.93653776	20.536701	13.2641613	1.23547921	16.7173446	88.0098412	3.72908325	8.26107556	87.4070374	8.12293094	4.4700317	97.7012422	2.2987578	2.25048455	25.719397	55.3188988	16.7112196
KI1.DG	25.5259073	32.3399312	17.0841167	9.06779055	15.9822542	68.8554773	5.41554802	25.7289746	13.3816032	2.46785452	21.6909271	15.693384	5.07155728	25.7122422	7.81520666	0.02810651	8.13911854	98.2732261	0.9834498	0.74332407	87.5885667	8.19787115	4.2135622	99.6383294	0.36167059	0.92424276	20.0177065	57.1732209	21.8848298
KI2.DG	37.3432231	21.4171185	6.04853538	9.04649332	26.1446297	66.7048089	19.6606756	13.6345154	5.36142193	0.51291983	16.1886568	19.0519135	1.17185933	18.3381177	15.2652881	1.84530243	22.2645205	84.0942653	11.4008058	4.50492889	88.9658069	5.45156297	5.5826301	98.3318264	1.66817356	1.09777258	11.0541562	66.050363	21.7977082
KI3.DG	33.2559879	12.6118499	25.6740226	10.9402557	17.5178839	68.8970034	16.6468674	14.4561292	11.927046	2.91771632	22.567979	13.9320335	3.87177381	26.1927196	6.05976643	0.93557025	11.5953951	95.5777539	2.06736835	2.35487771	88.8818179	7.4414642	3.67671786	99.0695671	0.93043293	1.06505624	20.21659	57.7971653	20.9211884
KO1.DG	20.005983	13.6240355	50.3886231	4.95387041	11.0274881	65.8987112	6.40831976	27.692969	15.1134046	5.96600359	25.7262487	10.9672216	5.15436701	28.279615	4.03719708	0.40566737	4.35027512	94.3995833	2.10515507	3.4952616	90.0155833	7.92875407	2.05566259	99.2518371	0.74816289	3.14800086	12.1855169	64.3613741	20.3051081
KO2.DG	18.3313386	16.1909463	49.3702924	5.19429225	10.9131304	66.5206328	5.94141918	27.5379481	11.1792723	2.85176979	18.0021517	24.6489982	3.68033152	27.40077	4.09284208	0.13460635	8.00925806	92.3386408	6.27530464	1.38605461	87.3902007	8.58462315	4.0251762	99.7561995	0.24380054	1.2454068	11.773204	69.0241287	17.9572606
WTI1.DG	24.0064867	32.8252493	24.3327948	7.71594082	11.1195285	70.4946493	6.17957479	23.3257759	12.5534417	4.17873056	23.0635475	17.8856805	4.37652868	29.4878932	4.6836012	0.07526015	3.69531656	97.011411	1.4906812	1.49790781	88.9734793	8.58398851	2.44253219	99.7807987	0.21920126	0.8384488	15.5669378	67.6314587	15.9631546
WTI2.DG	36.1978987	16.8543482	10.7388579	14.1607274	21.5981677	85.4520113	2.91233448	11.6356542	9.53144567	1.1073305	29.8002247	13.5569228	3.18736339	27.4078997	6.94954804	0.94832452	7.51094058	88.5851932	9.17505749	2.23974927	90.8704155	6.5924261	2.5371584	99.095393	0.90460701	0.78818505	25.3255038	56.5284524	17.3578587
WTO1.DG	31.6177239	11.1381474	27.34287	10.0698054	19.8314532	63.6393314	6.75638279	29.6042859	11.7773478	2.99584663	18.9141589	12.7638937	2.88607784	30.9903229	8.00840389	0.23272767	11.4312208	88.0224001	0.68984299	1.28775691	90.0834471	6.22623634	3.69031651	99.3954362	0.6045638	1.26136325	13.0626223	65.36779	20.3082245
WTO2.DG	36.2113	10.571091	6.73110459	14.6657082	31.8207962	64.1823464	17.3018683	18.5157853	9.96663561	1.42012544	30.2629848	10.2574424	1.38302708	31.8047313	4.63961646	0.43152301	9.83391377	99.784678	0.11153532	0.10378664	90.3474725	4.44281666	5.20971086	99.2897623	0.71023772	0.40345454	5.59341386	70.8338371	23.1692945
WTO3.DG	36.6094422	12.5866009	16.5984768	11.5633008	22.6421792	84.7948439	2.76618796	12.4389682	11.8921593	1.65001446	17.2269641	18.9441258	3.28509373	28.2137387	7.1386793	0.67187512	10.9773494	95.9577205	1.5940452	2.44823426	88.7632784	6.76672959	4.46999198	98.8872719	1.1127281	0.79434075	23.0443457	55.6828218	20.4784917
KI1.Cb	31.8980662	19.7056126	20.0360377	10.6513705	17.708913	69.700792	4.00450023	26.2947077	18.5640131	10.4189285	18.8736817	10.5649733	12.6381229	12.8946765	11.2279723	0.75695543	4.06067634	99.7130041	0.1691113	0.11788463	87.3087517	9.7120039	2.97924436	99.7889547	0.21104534	0.25714359	24.6733596	61.5651803	13.5043166
KI2.Cb	31.3353469	22.567817	15.0543233	10.0476553	20.9948574	68.5941456	3.89591327	27.5099411	20.641061	8.73811744	18.2636415	11.6226457	11.7931129	15.0654112	9.76341113	0.1762858	3.93631331	99.51799	0.25988877	0.22212119	73.7323077	17.9599956	8.30769671	99.8163593	0.18364072	0.20510649	21.2274583	65.4813572	13.086078
KI3.Cb	36.7184002	19.8157927	10.3647829	11.9457592	21.1552649	69.2028223	4.1567295	26.6404482	20.5044717	10.4158499	19.8803031	11.0002111	9.84428032	14.6932737	9.6314645	0.12535597	3.90478971	99.7638338	0.16652228	0.06964389	87.3585134	8.57504598	4.06644059	99.8985554	0.10144457	0.47658203	23.0477641	63.3180771	13.1575768
KO1.Cb	31.3728438	6.91545779	13.2013198	8.59597811	39.9144005	55.4359569	5.31807698	39.2459662	16.828371	6.0451983	27.6331471	10.1638831	1.70817879	16.7630247	16.0306129	0.15716838	4.67041571	98.6660634	0.29780352	1.0361331	92.8849194	3.83416208	3.28091853	99.4135932	0.58640683	0.7019352	4.27335927	49.0356864	45.9890191
KO2.Cb	34.6303022	32.0843205	8.03010663	8.26051207	16.9947586	69.6422426	4.35491379	26.0028436	22.6846631	11.6900049	17.1152897	8.31344939	8.78551282	14.0524162	10.4804857	0.07393656	6.80424161	99.6809462	0.23815323	0.08090055	87.6419318	9.09003065	3.26803755	99.812692	0.18730802	0.56278665	17.0758491	69.3059931	13.0553712
WTI1.Cb	18.0693267	12.2451628	54.3342955	5.44327781	9.9079371	77.9525054	2.09790204	19.9495925	23.9042585	9.77848156	18.5033827	11.6201607	16.3968529	7.19214391	6.92260264	3.25901519	2.42310198	99.5910302	0.19743468	0.2115351	88.9826945	9.3613308	1.65597474	99.7609484	0.23905157	0.2677286	33.2219382	55.6239644	10.8863689
WTI2.Cb	40.6792307	25.814875	5.00819032	9.51995101	18.9777529	69.6233323	3.99703673	26.3796309	21.9621419	10.1261355	14.1977802	10.3336557	15.9149104	13.6270763	9.48204053	0.06091413	4.2953454	99.7414595	0.13648099	0.12205948	79.1409342	14.9157751	5.94329064	99.7969439	0.20305612	0.2420773	35.0860975	47.2909314	17.3808938
WTO1.Cb	33.3775769	19.9327539	15.9734466	10.8432518	19.8729708	71.74297	4.18292872	24.0741013	22.0092724	9.40789206	18.124337	9.86259675	13.2580544	13.2891002	9.37484992	0.89997877	3.77391854	99.7026187	0.23363792	0.06374333	87.6252648	10.0550647	2.31967052	99.8060199	0.1939801	0.21814076	28.342587	58.9740132	12.465259
WTO2.Cb	33.1784266	15.603322	9.9333802	13.8349816	27.4498895	67.7012356	4.18531027	28.1134541	20.6460204	7.72611263	26.6740226	9.89444645	5.81481967	18.2349332	6.50602922	0.05945929	4.44415653	99.3721218	0.10496831	0.52290985	91.59237	6.36633237	2.0412976	99.8131302	0.18686981	0.868535	36.805202	32.1686584	30.1576046
WTO3.Cb	20.1719105	10.7704469	22.2574988	21.993088	24.8070557	68.8484273	2.45673267	28.69484	20.8264263	20.3188898	17.5379899	7.24652711	11.4239231	12.3838249	6.58504131	0.06534507	3.61203258	99.5600034	0.17211493	0.26788168	86.8414072	11.350337	1.80825578	99.8178665	0.18213351	0.83279411	33.2009652	18.8252045	47.1410362
