# Supplementary material for: Spatially resolved mapping of histones reveals selective neuronal response in Rett syndrome
Source: FEBS J. 2026 Apr 1;293(19):5814–36. doi: 10.1111/febs.70534 (PMC13628532; doi:10.1111/febs.70534)
Supplement: Supplementary file 2 [file FEBS-293-5814-s003.pdf]

Protein names	Sequence	Unique.Group	KI1.CA	KI1.Cb	KI1.DG	KI2.CA	KI2.Cb	KI2.DG	KI3.CA	KI3.Cb	KI3.DG	KO1.CA	KO1.Cb	KO1.DG	KO2.CA	KO2.Cb	KO2.DG
Total H2A	AGLQFPVGR	no	13.38194958	5.109335957	14.26420688	12.11381662	15.3264497	12.70918149	13.57344923	15.57056253	13.07401054	11.94868236	12.96855473	12.9448841	14.28008863	15.66617805	13.8232135
Total H2B	LLLPGELAKHA	no	9.755312769	13.1614557	11.18650877	8.677435417	13.4506706	10.19591344	9.648144238	13.53263258	10.18396009	8.363962534	12.62211166	10.05259675	10.3221855	14.08465456	11.08639853
Total H3	YRPGTVALR	no	12.03054006	14.13745946	13.55232063	10.09100461	13.96569772	12.03198653	12.94413194	14.52653402	11.9813536	11.09471003	11.90086446	11.87369989	13.09043678	14.68072281	12.81772751
Total H4	ISGLIYEETR	yes	12.76492499	14.59409375	13.65406038	11.33505336	14.47082544	12.12003569	12.91878536	14.96366459	11.74647916	10.93947756	12.09592167	11.84810962	13.39558985	15.07077615	12.91882269
Total H1	GTGASGSFKL	no	4.293225751	6.704249864	5.290532069	3.578438928	7.533639572	5.636334473	5.051937731	8.142127222	4.124297037	4.243488039	8.519546755	4.996951716	5.572049463	8.741321334	3.918813432
Histone H1.0	LVTTGVLKQT	yes	7.66010429	7.917043711	7.521913005	6.32781855	8.408673083	7.603453765	6.978243276	9.057075592	6.641493813	4.605153453	9.894498036	5.587965808	7.999314744	9.8696596	6.642152989
Histone H2A.V;Histone H2A.	GDEELDSLIK	yes	6.610439584	7.397914191	3.219906139	5.105293919	7.960640785	7.297777756	5.224409555	8.25117917	6.824069907	3.141349815	7.907315086	2.820854008	6.061291687	7.810692514	6.298746439
Histone H2A.J	VTIAQGGVLP	yes	7.292521207	5.079612635	7.250174142	4.909943037	1.981521301	5.75192692	6.067169975	6.071946634	5.749908576	5.144912289	1.612439064	4.963423503	7.442237556	6.377390147	6.906602752
Histone H2A type 2-B	HLQLAVR	yes	6.919881798	11.37329835	8.353371458	5.337374223	10.96812742	6.726011971	7.291201694	10.80190587	7.125875735	6.400883632	8.700451549	7.214745672	7.857186043	10.69882337	7.730161014
Core histone macro-H2A.1	GVTIASGGVLF	yes	4.464600724	5.780131177	5.612712324	4.233186886	6.543949245	5.424139889	2.590834259	6.057042213	5.645563277	4.297200778	6.064125359	5.605411526	4.223493234	6.31119036	1.998557682
Core histone macro-H2A.2	IHPELLAKKR	yes	6.278209812	1.516929319	7.478883569	6.461066446	9.132247548	5.017349967	6.489840845	8.844901539	5.10877465	6.111240521	7.275217561	6.256469549	6.866267279	11.33659565	7.077164141
Histone H2B type 1-A	ESYSIYIYKVLK	yes	4.820248064	2.143266798	7.587884655	2.505801347	8.66464249	8.350031467	2.14609449	9.093282464	4.939704694	4.185517233	4.473139502	4.789997238	6.456374673	1.708940643	7.544198656
Histone H2B type 2-B	PDPAKSAPAP	yes	6.392664659	7.797188664	8.000726448	3.954092747	8.761998461	5.817901129	2.099002472	9.454441061	6.507000621	4.969829108	6.213190918	8.200505298	7.705816341	10.82825537	8.560987729
Histone H2B type 3-B	PDPSKSAPAPK	yes	2.113242016	2.82440212	3.868822974	2.955354765	5.527650599	2.769472183	2.013335149	5.814691495	1.692688319	2.400963712	6.549737069	4.342397746	2.467091547	6.363560749	4.830777454
Histone H2B type 1-M	PEPTKSAPAPK	yes	3.154258334	5.758187685	5.393222725	2.204823244	6.848426168	2.523516724	2.882614002	6.224331219	1.977006199	2.065357615	2.319657104	1.571882864	1.993854731	4.544293831	4.873538041
Histone H1.0;Histone H1.0,	VGENADSQIKI	yes	6.866888859	9.418730552	7.134050514	4.713038777	9.427545656	6.29484422	6.286149084	7.628723966	6.226412179	5.570863989	1.587469734	5.699216734	6.405669975	10.42356093	6.421836129
Histone H1.1	SETAPVAQAA	yes	3.302737428	6.229895365	3.210595759	2.569209853	6.739556224	3.472629463	2.435168876	7.381199141	2.568037839	2.570707921	8.602097543	2.681999732	2.85252384	8.233247721	2.564421174
Histone H1.2	SEAAPAAPAA	yes	2.241867729	1.832731252	2.542630998	2.012378322	2.699112961	2.42487078	3.964428772	5.485253314	2.816719668	2.971627699	8.032026891	3.323883993	3.549694168	6.340458508	3.618914703
Histone H1.3	SETAPAAPAA	yes	6.294863811	7.764290834	7.941034638	3.022741415	8.314346252	7.459664437	5.911062449	8.657428132	7.178083685	4.887659757	9.414969335	6.277377887	6.492970618	9.478266484	7.380604208
Histone H1.4	SETAPAAPAA	yes	7.85980238	9.5381493	8.435877254	4.309619932	9.702278313	7.198274352	7.924173198	10.29293476	6.930184271	5.787899732	9.269969836	5.157004079	8.265366652	10.91410905	7.540299256
Histone H1.5	SETAPAETAA	yes	3.849375916	7.393195828	5.794481335	3.220195524	7.713637833	4.110659971	4.112493007	8.328175415	4.485542807	2.948207691	7.645950883	3.90487951	2.345381753	8.993449569	5.013870834

Protein names	WTI1.CA	WTI1.Cb	WTI1.DG	WTI2.CA	WTI2.Cb	WTI2.DG	WTO1.CA	WTO1.Cb	WTO1.DG	WTO2.CA	WTO2.Cb	WTO2.DG	WTO3.CA	WTO3.Cb	WTO3.DG
Total H2A	12.68559116	6.081078418	13.75959282	13.00900664	15.32467932	13.14909755	12.13026237	5.043863649	13.67554592	11.89059745	14.02043244	14.98393769	12.52285914	15.36921017	13.21266279
Total H2B	9.089586586	13.16868629	10.76793105	9.323059724	12.77510071	9.630787573	8.299800399	13.32083015	10.95250056	9.099796896	12.93801005	11.18111598	9.558467336	12.60351448	10.14456038
Total H3	11.80501092	13.81013643	12.79105258	11.63252923	14.35816517	12.07916947	10.95219878	14.17019925	12.58943744	11.09028543	12.84367311	13.72353373	11.67771801	10.29576641	12.68783282
Total H4	11.86101423	14.27970528	12.74877935	11.83662776	14.82006413	12.0633085	11.06353595	14.71263774	12.65382065	10.96265722	13.31986345	14.25953054	11.65605811	14.41598414	12.44874749
Total H1	4.435334148	7.669518934	5.097135067	3.783160044	7.044378389	4.966267578	3.906146666	8.046202804	5.471167278	4.612481551	9.773179702	7.44187815	5.191645903	2.473906357	3.497308696
Histone H1.0	6.670333009	7.697992172	6.874433241	7.055866053	8.30358177	6.901768724	4.793429911	7.901229585	6.040944523	5.811690994	9.966965733	9.467586728	7.240094522	7.35222754	6.491140786
Histone H2A.V;Histone H2A.	2.335588801	8.143484892	2.184841018	5.952741067	7.342904441	5.751298211	2.652743184	8.325765912	6.668741521	1.499498373	9.077279814	8.609234057	2.743101751	6.728653328	6.140928416
Histone H2A.J	5.714777869	5.171550868	6.353556267	6.326221498	5.33234123	5.70389416	4.487366244	2.895124949	6.709315313	5.524883654	6.094298401	7.24163433	6.167273482	1.643270387	5.896577469
Histone H2A type 2-B	6.638938354	5.043264309	7.451104571	6.383361213	10.78380525	6.611652331	6.269700376	11.5165786	7.881889535	6.387745207	9.611242427	8.055388915	6.887272254	4.126391745	7.112425069
Core histone macro-H2A.1	3.176162018	4.94796992	5.445269728	3.910338511	5.503263232	4.618254293	3.457935269	5.201753554	5.984481849	3.760959717	6.505590156	2.605602501	4.515729614	5.099481264	5.258558547
Core histone macro-H2A.2	5.798534733	8.708740153	7.175611147	5.816153464	9.136636216	4.733971024	6.559409414	9.011650034	6.696975537	5.827953348	9.172641468	5.423403547	3.707209831	9.178831944	6.819007773
Histone H2B type 1-A	2.697742029	7.868676134	3.692179717	2.160920197	2.038897847	6.790560729	2.310869457	8.446712147	2.372356757	2.702822291	9.756399523	8.855208903	4.61320821	7.141976215	3.150803302
Histone H2B type 2-B	5.838323454	8.086402876	8.006804744	5.837647345	8.555100188	5.575867917	6.413804542	9.814473347	7.674445239	5.554273431	9.268085449	8.33174978	5.793355296	8.535033522	6.349064318
Histone H2B type 3-B	2.733544317	2.410432619	4.106091837	2.961451802	5.195884549	2.880913305	1.978418976	5.538992307	3.915704831	2.280109851	6.487242934	4.620788661	2.245694606	5.672415501	2.876666338
Histone H2B type 1-M	2.395575876	5.35342708	4.607652824	2.655816445	5.119350243	2.221645781	1.891577374	1.835777054	2.239868777	2.620560986	7.273799787	6.501128695	2.519509766	2.125122513	3.658547151
Histone H1.0;Histone H1.0,	6.08079773	9.802352349	7.746217637	6.512224895	9.553723145	6.429978511	5.000988721	9.248407827	5.280184677	4.837242013	9.025166412	8.391178829	4.501302901	1.558789637	5.981243918
Histone H1.1	2.233705232	5.883896424	3.607893855	3.558212344	5.808854037	2.815177327	1.948369353	6.887273447	2.711099027	1.630869651	9.453290582	5.876856939	1.792811991	2.03272773	2.589526999
Histone H1.2	3.295951038	4.761307923	3.200075421	3.015838138	3.63441191	1.866942609	3.246831081	1.804238857	3.469306109	2.481916098	6.467032699	4.730398598	2.167914873	2.543660183	2.750259489
Histone H1.3	5.083182748	7.519988393	7.46121834	5.814387105	7.58534627	6.937460018	2.885697119	8.276945979	7.172530289	4.946696322	9.954297403	9.540080199	5.693405828	7.775005537	7.259006791
Histone H1.4	6.817148943	9.466814971	4.877968277	6.953232573	8.231305126	5.964326386	5.492042659	9.799084068	7.191954213	6.191498038	11.22409938	9.180087557	6.972363376	8.014798476	4.665009024
Histone H1.5	2.347942319	6.735280725	5.005877771	3.825311673	7.567415864	2.319370776	1.575386828	7.178947556	1.93629005	1.944049426	8.88351686	6.833199756	3.941610104	7.105063693	2.878098391
