# Supplementary material for: Spatially resolved mapping of histones reveals selective neuronal response in Rett syndrome
Source: FEBS J. 2026 Apr 1;293(19):5814–36. doi: 10.1111/febs.70534 (PMC13628532; doi:10.1111/febs.70534)
Supplement: Supplementary file 3 [file FEBS-293-5814-s002.docx]

**Spatially resolved mapping of histones reveal selective neuronal response in Rett syndrome**

Frederike Schäfer^1^, Guiseppa de Rocco^2,3^, Moritz Völker-Albert^4^, Ignasi Forne^5^, Nicoletta Landsberger^2,3^, Axel Imhof^1,5^*, Shibojyoti Lahiri ^1,5^*

^1^ Biomedical Center Munich, Department of Molecular Biology, Faculty of Medicine, Ludwig-Maximilians Universität München, Planegg-Martinsried, Germany

^2^ San Raffaele Rett Research Unit, Neuroscience Division, IRCCS San Raffaele Scientific Institute, Milan, Italy

^3^ Department of Medical Biotechnology and Translational Medicine, Faculty of Medicine, University of Milan, Segrate, Milan, Italy

^4^ MoleQlar Analytics GmbH, Rosenheimer Straße 141h, 81671 München, Germany

^5^ Biomedical Center Munich, Protein Analysis Unit (ZfP), Faculty of Medicine, Ludwig-Maximilians Universität München, Planegg-Martinsried, Germany

*Corresponding authors: imhof@lmu.de, shibojyoti.lahiri@med.uni-muenchen.de

**SUPPLEMENTAL INFORMATION**

**SUPPLEMENTAL FIGURES**

**Supplemental Figure S1: Histone proteoforms are sufficient to separate regions of interest in RTT brains. A.** Loadings plot of PCA analysis of MSI data. Highly contributing masses (histones are major contributors) are highlighted in red (Mass list: Supplemental file S1, Sheet: Loadings plot). *Inset*: Spatial distribution pattern of all the aforementioned masses (Scale bar = 5 mm). **B**. Associated functions to chosen regions of interest that are affected in RTT. **C.** PCA of LC-MS/MS data of histone peptides. Even these reduced features are sufficient to separate the neuronal population DG, CA and Cb.

**Supplemental Figure S2: Exclusion of potential confounding factors influencing histone abundances. A.** Proportion of acid-extracted proteins with stable expression in *Mecp2^-/y^* and *Mecp2^Y120D/y^*. **B.** log2 fold change of cell-specific control proteins between WT and *Mecp2^-/y^* / *Mecp2^Y120D/y^***. C.** Total DNA amount in ng per neuronal population.

**Supplemental Figure S3: Alteration of the H3 pool, H2A/B isoform coverage maps and comparison of histone abundances with respective mRNA. A.** Relative abundance of H3.1/3.2 and H3.3 in WT and *Mecp2^-/y^* / *Mecp2^Y120D/y^* brains. **B.** Position within protein of detected peptides in H2A and H2B. Unique peptides are color-coded, shared peptides are grey. **C.** Comparison of our proteomics data with publicly available total RNA-seq data ^62-67^. Indicated as log2 fold change between WT and *Mecp2^-/y^.*

**Supplemental Figure S4: Alteration of specific histone modifications and corresponding writers/erasers in *Mecp2^-/y^* along with H1 variant coverage maps**. **A.** Fold change between WT and *Mecp2^-/y^* of histone modifying enzymes detected in whole proteomic extract and associated with Mecp2 function or deposition of quantified histone marks. **B.** Relative abundance [%] of modified peptides to the overall peptide in WT and *Mecp2^-/y^* brains. The difference between percentual contributions is displayed as ratio (*Mecp2^-/y^*/WT). **C.** Relative abundance of H3.1/3.2 and H3.3 in WT and *Mecp2^-/y^* brains. **D.** Position within protein of detected peptides in H1 variants. Unique peptides are color-coded, shared peptides are grey.

**Supplemental Figure S5: Alteration of histone proteoforms (including linker histone H1) in *Mecp2^Y120D/y^* and quantification of Mecp2**. **A.** Total H3/H4 acetylation levels in neuronal populations. Displayed as log2 fold change between WT and *Mecp2^Y120D/y^***. B.** Relative abundance [%] of modified peptides to the overall peptide in WT and *Mecp2^Y120D/y^* brains. The difference between relative contributions is displayed as ratio (*Mecp2^Y120D/y^*/WT). **C.** Relative contribution of unmodified peptides to the overall peptide as a ratio between WT and *Mecp2^Y120D/y^*. **D.** Fold change between WT and *Mecp2^Y120D/y^* of histone modifying enzymes detected in whole proteomic extract and associated with Mecp2 function or deposition of quantified histone marks. **E.** log2FC of total histone H1 in WT and *Mecp2^Y120D/y^*. **F.** Quantification of detected Mecp2 peptides in *Mecp2^-/y^* and *Mecp2^Y120D/y^* brains and their respective WT littermates.

SUPPLEMENTAL TABLES

**Supplemental Table S1**. MSI results. Masses contributing prominently to the loadings plot in Fig. S1A (highlighted) are listed.

Centroid [m/z]

5657.978

5677.601

6904.771

7008.921

7028.544

7667.035

7695.714

11315.338

11357.603

11393.829

13789.301

14014.207

14050.434

15299.491

15328.925

15353.075

**Supplemental Table S2.** MSI results. Detected masses used to plot the heatmap in Fig 1D that were assigned to theoretical histone masses based on previous work^52^.

**Supplemental Table S3.** Information about compaction ability scoring used for Fig. 3C (Sheets: Scoring system, DG, CA, Cb and compaction). 1 = weakest effect on chromatin compaction; 6 = highest effect on chromatin compaction. Based on previous work^68^.

| Peptide | Chromatin affinity | Compaction ability | Expression in differentiated cells | Combined Score |
| --- | --- | --- | --- | --- |
| H1.0 | Intermediate | High | High | 4 |
| H1.1 | Low | Weak | Low | 1 |
| H1.2 | Intermediate | Weak | High | 2 |
| H1.3 | Intermediate | Intermediate | Low | 3 |
| H1.4 | High | High | High | 6 |
| H1.5 | High | High | Low | 5 |

**Supplemental Table S4.** Information about compaction ability scoring used for Fig. 3. Compaction scoring in DG, CA, Cb.

| Peptide | KO_DG | KI_DG | KO_CA | KI_CA | KO_Cb | KI_Cb |
| --- | --- | --- | --- | --- | --- | --- |
| H1.0 | -0.4903427 | -0.5363291 | 1.20842244 | -0.3411524 | -0.6052726 | -0.8530377 |
| H1.1 | -1.1026172 | -0.1277812 | 0.92093222 | -0.1269201 | 2.29324205 | 0.93717501 |
| H1.2 | -0.1785887 | 0.06123147 | 0.62844025 | -0.4163363 | 3.58126545 | -0.8588274 |
| H1.3 | -1.161548 | 0.32692174 | 1.18171543 | -0.3725624 | 0.77786827 | 0.69268774 |
| H1.4 | -0.6636986 | 2.10029796 | 0.8079985 | -0.1873256 | 0.41271214 | 0.99539407 |
| H1.5 | 0.57684577 | 1.13427043 | 0.15977927 | 0.64072782 | 0.59719086 | 0.6603214 |

**Supplemental Table S5.** Histone coverage maps for all histone isoforms. Covered sequences are highlighted.

| Histone | Sequence |
| --- | --- |
| H2A type 2-B | MSGRGKQGGKARAKAKSRSSRAGLQFPVGRVHRLLRKGNYAERVGAGAPVYMAAVLEYLTAEILELAGNAARDNKKTRIIPRHLQLAVRNDEELNKLLGGVTIAQGGVLPNIQAVLLPKKTESHKPGKNK |
| H2A.Z | MAGGKAGKDSGKAKTKAVSRSQRAGLQFPVGRIHRHLKSRTTSHGRVGATAAVYSAAILEYLTAEVLELAGNASKDLKVKRITPRHLQLAIRGDEELDSLIKATIAGGGVIPHIHKSLIGKKGQQKTV |
| H2A.J | MSGRGKQGGKVRAKAKSRSSRAGLQFPVGRVHRLLRKGNYAERVGAGAPVYLAAVLEYLTAEILELAGNAARDNKKTRIIPRHLQLAIRNDEELNKLLGRVTIAQGGVLPNIQAVLLPKKTESQKVKSK |
| H2B type 2-B | MPDPAKSAPAPKKGSKKAVTKVQKKDGKKRKRSRKESYSVYVYKVLKQVHPDTGISSKAMGIMNSFVNDIFERIAGEASRLAHYNKRSTITSREIQTAVRLLLPGELAKHAVSEGTKAVTKYTSSK |
| H2B type  1-A | MPEVAVKGATISKKGFKKAVTKTQKKEGRKRKRCRKESYSIYIYKVLKQVHPDTGISSKAMSIMNSFVTDIFERIASEASRLAHYNKRSTITSREIQTAVRLLLPGELAKHAVSEGTKAVTKYTSSK |
| H2B type 3-B | MPDPSKSAPAPKKGSKKAVTKAQKKDGKKRKRGRKESYSIYVYKVLKQVHPDTGISSKAMGIMNSFVNDIFERIASEASRLAHYNKRSTITSREVQTAVRLLLPGELAKHAVSEGTKAVTKYTSSK |
| H2B type 1-M | MPEPTKSAPAPKKGSKKAVTKAQKKDGKKRKRSRKESYSVYVYKVLKQVHPDTGISSKAMGIMNSFVNDIFERIAGEASRLAHYNKRSTITSREIQTAVRLLLPGELAKHAVSEGTKAVTKYTSSK |
| Macro H2A.1 | MSSRGGKKKSTKTSRSAKAGVIFPVGRMLRYIKKGHPKYRIGVGAPVYMAAVLEYLTAEILELAGNAARDNKKGRVTPRHILLAVANDEELNQLLKGVTIASGGVLPNIHPELLAKKRGSKGKLEAIITPPPAKKAKSPSQKKPVAKKTGGKKGARKSKKKQGEVSKAASADSTTEGTPTDGFTVLSTKSLFLGQKLQVVQADIASIDSDAVVHPTNTDFYTGGEVGNTLEKKGGKEFVEAVLELRKKNGPLEVAGAAISAGHGLPAKFVIHCNSPVWGADKCEELLEKTVKNCLALADDRKLKSIAFPSIGSGRNGFPKQTAAQLILKAISSYFVSTMSSSIKTVYFMLFDSESIGIYVQEMAKLDAN |
| Macro H2A.2 | MSGRSGKKKMSKLSRSARAGVIFPVGRLMRYLKKGTFKYRISVGAPVYMAAVIEYLAAEILELAGNAARDNKKARIAPRHILLAVANDEELNQLLKGVTIASGGVLPRIHPELLAKKRGTKGKSETILSPPPEKRGRKAASGKKGGKKSKATKPRTSKKSKAKDSDKEGTSNSTSEDGPGDGFTILSSKSLVLGQKLSLTQSDISHIGSMRVEGIVHPTTAEIDLKEEIGKALEKAGGKEFLETVKELRKSQGPLEVAEAAVSQSSGLAAKFVIHCHIPQWGSDKCEEQLEETIKNCLSAAEDKKLKSVAFPPFPSGRNCFPKQTAAQVTLKAISAHFDDSSSSSLKNVYFLLFDSESIGIYVQEMAKLDTK |
| H3.1 | MARTKQTARKSTGGKAPRKQLATKAARKSAPATGGVKKPHRYRPGTVALREIRRYQKSTELLIRKLPFQRLVREIAQDFKTDLRFQSSAVMALQEACEAYLVGLFEDTNLCAIHAKRVTIMPKDIQLARRIRGERA |
| H3.2 | MARTKQTARKSTGGKAPRKQLATKAARKSAPATGGVKKPHRYRPGTVALREIRRYQKSTELLIRKLPFQRLVREIAQDFKTDLRFQSSAVMALQEASEAYLVGLFEDTNLCAIHAKRVTIMPKDIQLARRIRGERA |
| H3.3 | MARTKQTARKSTGGKAPRKQLATKAARKSAPSTGGVKKPHRYRPGTVALREIRRYQKSTELLIRKLPFQRLVREIAQDFKTDLRFQSAAIGALQEASEAYLVGLFEDTNLCAIHAKRVTIMPKDIQLARRIRGERA |
| H4 | MSGRGKGGKGLGKGGAKRHRKVLRDNIQGITKPAIRRLARRGGVKRISGLIYEETRGVLKVFLENVIRDAVTYTEHAKRKTVTAMDVVYALKRQGRTLYGFGG |
| H1.0 | MTENSTSAPAAKPKRAKASKKSTDHPKYSDMIVAAIQAEKNRAGSSRQSIQKYIKSHYKVGENADSQIKLSIKRLVTTGVLKQTKGVGASGSFRLAKGDEPKRSVAFKKTKKEVKKVATPKKAAKPKKAASKAPSKKPKATPVKKAKKKPAATPKKAKKPKVVKVKPVKASKPKKAKTVKPKAKSSAKRASKKK |
| H1.1 | MSETAPVAQAASTATEKPAAAKKTKKPAKAAAPRKKPAGPSVSELIVQAVSSSKERSGVSLAALKKSLAAAGYDVEKNNSRIKLGLKSLVNKGTLVQTKGTGAAGSFKLNKKAESKAITTKVSVKAKASGAAKKPKKTAGAAAKKTVKTPKKPKKPAVSKKTSKSPKKPKVVKAKKVAKSPAKAKAVKPKASKAKVTKPKTPAKPKKAAPKKK |
| H1.2 | MSEAAPAAPAAAPPAEKAPAKKKAAKKPAGVRRKASGPPVSELITKAVAASKERSGVSLAALKKALAAAGYDVEKNNSRIKLGLKSLVSKGILVQTKGTGASGSFKLNKKAASGEAKPQAKKAGAAKAKKPAGAAKKPKKATGAATPKKAAKKTPKKAKKPAAAAVTKKVAKSPKKAKVTKPKKVKSASKAVKPKAAKPKVAKAKKVAAKKK |
| H1.3 | MSETAPAAPAAPAPVEKTPVKKKAKKTGAAAGKRKASGPPVSELITKAVAASKERSGVSLAALKKALAAAGYDVEKNNSRIKLGLKSLVSKGTLVQTKGTGASGSFKLNKKAASGEAKPKAKKAGAAKAKKPAGAAKKPKKATGAATPKKTAKKTPKKAKKPAAAAGAKKVSKSPKKVKAAKPKKAAKSPAKAKAPKAKASKPKASKPKATKAKKAAPRKK |
| H1.4 | MSETAPAAPAAPAPAEKTPVKKKARKAAGGAKRKTSGPPVSELITKAVAASKERSGVSLAALKKALAAAGYDVEKNNSRIKLGLKSLVSKGTLVQTKGTGASGSFKLNKKAASGEAKPKAKRAGAAKAKKPAGAAKKPKKAAGTATAKKSTKKTPKKAKKPAAAAGAKKAKSPKKAKATKAKKAPKSPAKAKTVKPKAAKPKTSKPKAAKPKKTAAKKK |
| H1.5 | MSETAPAETAAPAPVEKSPAKKKTTKKAGAAKRKATGPPVSELITKAVSASKERGGVSLPALKKALAAGGYDVEKNNSRIKLGLKSLVSKGTLVQTKGTGASGSFKLNKKAASGEAKPKAKKTGAAKAKKPAGATPKKPKKTAGAKKTVKKTPKKAKKPAAAGVKKVAKSPKKAKAAAKPKKAAKSPAKPKAVKSKASKPKVTKPKTAKPKAAKAKKAVSKKK |

**Supplemental Table S6.** Results of LIMMA statistical analysis between WT and mutant groups.

| Variable | Contrast | LogFC  DG | adjPValue DG | LogFC  CA | adjPValue CA | LogFC  Cb | adjPValue Cb |
| --- | --- | --- | --- | --- | --- | --- | --- |
| H2A | KO - WTO | -0.57 | 0.45 | 0.93 | 0.27 | -0.38 | 0.94 |
| H2A | KI - WTI | -0.11 | 0.89 | 0.18 | 0.83 | 1.30 | 0.78 |
| H2B | KO - WTO | -0.19 | 0.76 | 0.36 | 0.62 | 0.40 | 0.41 |
| H2B | KI - WTI | 0.32 | 0.60 | 0.15 | 0.83 | 0.41 | 0.40 |
| H3 | KO - WTO | -0.65 | 0.36 | 0.85 | 0.41 | 0.85 | 0.53 |
| H3 | KI - WTI | 0.09 | 0.90 | -0.03 | 0.98 | 0.13 | 0.93 |
| H4 | KO - WTO | -0.74 | 0.40 | 0.94 | 0.29 | -0.57 | 0.55 |
| H4 | KI - WTI | 0.10 | 0.91 | 0.49 | 0.57 | 0.13 | 0.89 |
| H1 | KO - WTO | -1.16 | 0.29 | 0.41 | 0.70 | 1.31 | 0.23 |
| H1 | KI - WTI | 0.30 | 0.78 | 0.14 | 0.89 | 0.30 | 0.77 |
| Histone H2A type 2-B | KO - WTO | -0.21 | 0.73 | 0.61 | 0.41 | 1.28 | 0.64 |
| Histone H2A type 2-B | KI - WTI | 0.37 | 0.55 | 0.01 | 0.99 | 3.13 | 0.27 |
| H2A.Z | KO - WTO | -2.58 | 0.22 | 2.30 | 0.14 | -0.18 | 0.80 |
| H2A.Z | KI - WTI | 1.81 | 0.37 | 1.50 | 0.31 | 0.13 | 0.86 |
| H2A.J | KO - WTO | -0.68 | 0.42 | 0.90 | 0.40 | 0.45 | 0.84 |
| H2A.J | KI - WTI | 0.22 | 0.79 | 0.07 | 0.95 | -0.87 | 0.69 |
| macro-H2A.1 | KO - WTO | -0.81 | 0.57 | 0.35 | 0.61 | 0.59 | 0.28 |
| macro-H2A.1 | KI - WTI | 0.53 | 0.71 | 0.22 | 0.74 | 0.90 | 0.11 |
| macro-H2A.2 | KO - WTO | 0.35 | 0.75 | 1.12 | 0.21 | 0.18 | 0.94 |
| macro-H2A.2 | KI - WTI | -0.09 | 0.94 | 0.60 | 0.48 | -2.42 | 0.37 |
| Histone H2B type 1-A | KO - WTO | 1.37 | 0.58 | 2.11 | 0.12 | -5.36 | 0.10 |
| Histone H2B type 1-A | KI - WTI | 1.72 | 0.49 | 0.73 | 0.56 | 1.68 | 0.56 |
| H2B type 2-B | KO - WTO | 0.93 | 0.40 | 0.42 | 0.77 | -0.69 | 0.63 |
| H2B type 2-B | KI - WTI | -0.02 | 0.99 | -1.69 | 0.26 | 0.35 | 0.80 |
| Histone H2B type 3-B | KO - WTO | 0.78 | 0.37 | 0.27 | 0.40 | 0.56 | 0.65 |
| Histone H2B type 3-B | KI - WTI | -0.72 | 0.41 | -0.49 | 0.15 | 0.92 | 0.46 |
| Histone H2B type 1-M | KO - WTO | -0.91 | 0.64 | -0.31 | 0.39 | -0.31 | 0.86 |
| Histone H2B type 1-M | KI - WTI | -0.12 | 0.95 | 0.22 | 0.54 | 1.04 | 0.57 |
| H1.0 | KO - WTO | -0.49 | 0.64 | 1.21 | 0.11 | -0.61 | 0.86 |
| H1.0 | KI - WTI | -0.54 | 0.61 | -0.34 | 0.62 | -0.85 | 0.81 |
| H1.1 | KO - WTO | -1.10 | 0.33 | 0.92 | 0.08 | 2.29 | 0.30 |
| H1.1 | KI - WTI | -0.13 | 0.91 | -0.13 | 0.78 | 0.94 | 0.66 |
| H1.2 | KO - WTO | -0.18 | 0.79 | 0.63 | 0.38 | 3.58 | 0.09 |
| H1.2 | KI - WTI | 0.06 | 0.93 | -0.42 | 0.55 | -0.86 | 0.64 |
| H1.3 | KO - WTO | -1.16 | 0.20 | 1.18 | 0.40 | 0.78 | 0.27 |
| H1.3 | KI - WTI | 0.33 | 0.70 | -0.37 | 0.78 | 0.69 | 0.33 |
| H1.4 | KO - WTO | -0.66 | 0.66 | 0.81 | 0.57 | 0.41 | 0.70 |
| H1.4 | KI - WTI | 2.10 | 0.20 | -0.19 | 0.89 | 1.00 | 0.37 |
| H1.5 | KO - WTO | 0.58 | 0.74 | 0.16 | 0.85 | 0.60 | 0.44 |
| H1.5 | KI - WTI | 1.13 | 0.51 | 0.64 | 0.47 | 0.66 | 0.39 |
| H3K9ac | KO - WTO | 0.83 | 0.16 | -0.70 | 0.56 | -0.51 | 0.84 |
| H3K9ac | KI - WTI | 0.05 | 0.92 | 1.25 | 0.32 | 0.05 | 0.98 |
| H3K27ac | KO - WTO | 0.00 | 1.00 | -0.93 | 0.51 | -0.50 | 0.70 |
| H3K27ac | KI - WTI | 0.36 | 0.71 | -1.64 | 0.31 | 0.04 | 0.97 |
| H3K9me3 | KO - WTO | 0.74 | 0.21 | -2.45 | 0.16 | -1.46 | 0.57 |
| H3K9me3 | KI - WTI | -0.41 | 0.46 | 0.97 | 0.55 | 0.10 | 0.97 |
| H3K27me3 | KO - WTO | 0.95 | 0.24 | -0.39 | 0.61 | -1.27 | 0.32 |
| H3K27me3 | KI - WTI | -0.24 | 0.75 | -0.32 | 0.69 | -0.27 | 0.82 |
| H3K18ac | KO - WTO | 0.08 | 0.95 | -1.90 | 0.05 | -0.52 | 0.67 |
| H3K18ac | KI - WTI | 1.45 | 0.27 | -0.12 | 0.88 | 0.18 | 0.88 |
| H3K23ac | KO - WTO | 0.78 | 0.20 | -0.69 | 0.50 | -0.73 | 0.62 |
| H3K23ac | KI - WTI | 0.00 | 1.00 | 0.57 | 0.58 | -0.07 | 0.96 |
| H3K36me1 | KO - WTO | 1.55 | 0.13 | 0.19 | 0.84 | -2.62 | 0.06 |
| H3K36me1 | KI - WTI | -0.68 | 0.47 | -0.77 | 0.47 | -0.79 | 0.51 |
| H3K36me2 | KO - WTO | 0.54 | 0.36 | -0.50 | 0.45 | -1.22 | 0.36 |
| H3K36me2 | KI - WTI | -0.59 | 0.32 | -0.83 | 0.27 | 0.23 | 0.86 |
| H3.1K36ac | KO - WTO | -0.14 | 0.96 | -1.91 | 0.59 | -1.80 | 0.44 |
| H3.1K36ac | KI - WTI | 0.16 | 0.95 | -0.82 | 0.82 | -1.09 | 0.64 |
| H3K79me2 | KO - WTO | -0.98 | 0.31 | -0.99 | 0.55 | -0.33 | 0.79 |
| H3K79me2 | KI - WTI | 0.77 | 0.41 | 0.99 | 0.55 | 0.20 | 0.88 |
| H3K27me2 | KO - WTO | 0.67 | 0.30 | -0.67 | 0.44 | -1.22 | 0.39 |
| H3K27me2 | KI - WTI | -0.68 | 0.30 | -0.70 | 0.46 | -0.06 | 0.97 |
| H3K56me1 | KO - WTO | 1.47 | 0.52 | -2.16 | 0.45 | -0.39 | 0.89 |
| H3K56me1 | KI - WTI | -0.04 | 0.98 | 0.08 | 0.98 | -0.96 | 0.74 |
| H4K20me2 | KO - WTO | 0.77 | 0.36 | -1.54 | 0.17 | 2.73 | 0.06 |
| H4K20me2 | KI - WTI | -0.29 | 0.72 | 0.30 | 0.77 | 0.24 | 0.85 |
| H4K20me3 | KO - WTO | 0.55 | 0.51 | -1.49 | 0.18 | 1.81 | 0.37 |
| H4K20me3 | KI - WTI | 0.12 | 0.89 | 0.62 | 0.55 | -0.13 | 0.95 |
